# Correlation between the clinical disability and T1 hypointense lesions’ volume in cerebral magnetic resonance imaging of multiple sclerosis patients: A systematic review and meta‐analysis

**DOI:** 10.1111/cns.13734

**Published:** 2021-10-03

**Authors:** Amir Valizadeh, Mana Moassefi, Elham Barati, Mohammad Ali Sahraian, Faezeh Aghajani, Mohammad‐Reza Fattahi

**Affiliations:** ^1^ Tehran University of Medical Sciences Tehran Iran

**Keywords:** demyelination, magnetic resonance imaging, multiple sclerosis, neurology, neurosciences

## Abstract

**Background:**

To evaluate the correlation between T1 hypointense lesions’ mean volume on cerebral MRI with disability level of patients with multiple sclerosis.

**Methods:**

We included studies testing the desired outcome in adult patients diagnosed with RRMS or SPMS. In Feb 2021, we searched PubMed, Embase, CENTRAL, and Web of Science to find relevant studies. All included studies were assessed for the risk of bias using a tailored version of the Quality in Prognosis Studies (QUIPS) tool. Extracted correlation coefficients were converted to the Fisher's z scale, and a meta‐analysis using a random‐effects model was performed on the results.

**Results:**

We included 27 studies (1919 participants). Meta‐analysis revealed a correlation coefficient of 0.32 (95% CI 0.26–0.37) between T1 hypointense lesions’ mean volume and EDSS score.

**Discussion:**

The correlation between T1 hypointense lesions’ mean volume and EDSS was interpreted as low to slightly moderate. The certainty of the evidence was judged to be high.

## Graphical abstract

Correlation coefficient; 95% Cl: 95% Confidence Intervals; Each red square indicates the effect size in one study, while the size of the square indicates the weight associated to that study in the meta‐analysis. Horizontal black lines represent the 95% Cl associated with each effect size. Black diamond represents the summary effect and its 95% Cl, while the red line represents the prediction intervals.

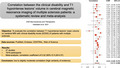

## 1 INTRODUCTION

### 1.1 Rationale

Multiple sclerosis (MS) is a chronic, disabling disease that targets patients in work productive years. Prognostic factors play important roles in evaluating the cost of the disease on both individual patients and the social economy. Since there is no exact method to predict MS progression confidently, four general MS phenotypes have been defined: clinically isolated syndrome (CIS), relapsing‐remitting MS (RRMS), secondary progressive (SPMS), and primary progressive (PPMS).^1^, ^2^ RRMS is the most common MS phenotype.

Magnetic resonance imaging (MRI) is a sensitive paraclinical test for diagnosis and assessment of disease progression in MS and is often used to evaluate therapeutic efficacy. White matter lesions on T2WI are hyperintense and could indicate several different histopathological changes such as edema, inflammation, demyelination, gliosis, and axonal loss.^3^ On the other hand, T1WI hypointense white matter lesions mostly correspond to axonal loss, white matter destruction, axonal loss, and irreversible clinical outcome.^4^, ^5^ As the role of neurodegeneration in the pathophysiology of MS has become more prominent, the formation and evolution of these lesions have been used to measure disease activity. These lesions result from an expansion of the extracellular space due to either an increase in water content or a deterioration of structural components.^6^ This reaction may be the consequence of tissue destruction or increased water influx through the impaired blood‐brain barrier. Some variations in these lesions have been identified. As Adusumilli et al. showed, using spin‐echo (SE) sequence images, these lesions can be classified based on the levels of the hypointensity to gray holes (less hypointense) and black holes (more hypointense).^7^ These classes are correlated with different clinical and cognitive measures.^7^, ^8^ Sahraian et al. define a black hole as “an area that is hypointense compared with the white matter in a T1WI and is concordant with a hyperintense lesion on a T2WI”.^5^ They also propose black holes can be divided into two groups: acute (when it coincides with a contrast‐enhancing lesion) and chronic or persistent (those lesions that appear hypointense in T1WI, but do not enhance after contrast injection). Some other authors consider a black hole persistent if it persists for more than 6 months after its first appearance on MRI.^4^, ^9^ The evolution of T1WI hypointense lesions is also of importance. While some remain unchanged during the time, some other convert to isointense lesions (probably due to extensive or partial remyelination).^4^ In a longitudinal study by van Waesberghe et al. on T1WI, 55% of hypointense lesions converted to isointense lesions by six months, while the rest remained unchanged.^10^ It is also worth mentioning that 25% of the isointense lesions in their patients converted to hypointense lesions after that time interval.

Truyen et al. were the first to describe an association between T1WI hypointense lesions and the clinical state of MS patients.^11^ In their study, when using MRI to evaluate the clinical disability, T1 hypointense lesion load showed greater cross‐sectional and longitudinal^11^ correlations with Expanded Disability Status Scale (EDSS)^12^ scores (a scale extensively used in studies for the assessment of disability for patients with MS) for patients with RRMS or SPMS than did T2 lesion load. In contrast, some other studies reported an absence of correlations between T1 hypointensity and EDSS in patients with SPMS^13^, ^14^, ^15^, ^16^, ^17^ and RRMS.^17^ O’Riordan et al. even reported a negative correlation between these measures.^16^

Considering the importance of these lesions and also considering the controversial findings of their association with the clinical disability of patients, in this review, we aimed to systematically assess the available findings regarding this matter up to this date.

### 1.2 Objectives

To evaluate the correlation between T1 hypointense lesions lesion mean volume on cerebral MRI with disability level of patients with RRMS or SPMS.

## 2 MATERIAL AND METHODS

Design and methods used for this review comply with CRD’s Guidance for Undertaking Reviews in Healthcare^18^ and are reported in line with Preferred Reporting Items for Systematic Reviews and Meta‐Analyses 2020 (PRISMA 2020).^19^

### 2.1 Eligibility criteria

Eligibility criteria were informed using the PICOTS system:

(P) Population: adult patients diagnosed with RRMS or SPMS, based on the McDonald criteria^2^ or Clinically Definite Multiple Sclerosis (CDMS) based on Poser criteria.^20^
(I) Index (Prognostic factor): T1 hypointense lesion mean volume on cerebral MRI.
(C) Comparator: not applicable.
(O) Outcome: disability measure using EDSS.
(T) Timing: measured at the same time MRI was performed (or with a time interval of less than a week in between).
(S) Setting: any.

### 2.2 Information sources

#### 2.2.1 Online databases

The search employed sensitive topic‐based strategies designed for each database with no time frame, language, or geographical restrictions. On the 10^th^ of February 2021, AV performed the search in the following databases:

- MEDLINE (PubMed)
- Embase
- Cochrane Central Register of Controlled Trials
- Web of Science (Core Collection)

#### 2.2.2 Citation searching

We also examined the forward and backward citations of the included studies on the 25^th^ of February 2021 using Scopus.

### 2.3 Search strategy

Our search was designed in line with PRISMA‐S guideline^21^ and is presented in (Appendix S1).

### 2.4 Selection process

AV and MM independently screened the titles and abstracts of identified studies for inclusion. Disagreements in this stage were resolved through discussion. Full text of potentially eligible studies was retrieved. Each study was included when both reviewers independently assessed it as satisfying the inclusion criteria from the full text. MF acted as arbiter in the event of disagreement following discussion.

### 2.5 Data collection process

Using a standardized form, AV and MM extracted the data independently. We resolved any disagreements by discussion. In cases in which PPMS or CIS patients were also included in a study, we included such studies only if CIS and PPMS patients consisted less than 15% of the study's sample size.

### 2.6 Data items

#### 2.6.1 Outcomes

The main outcome of interest was the correlation between the T1 hypointense lesions’ mean volume on cerebral MRI and the EDSS score of participants. Some studies reported measurements at multiple time points for the same participants. In these cases, we averaged the presented correlation coefficients using the formula presented by Alexander^22^:

$$r_{\mathrm{average}}=\;\frac{\sum (n_{i}‐1)}{\sum n_{i}‐k}\left\{r_{i}+\left[\frac{r_{i}\left(1‐r_{i}^{2}\right)}{2(n_{i}‐3)}\right]\right\}$$

#### 2.6.2 Other variables

Other variables of interest that we extracted from the included studies were the following:

- sample characteristics
- sample size
- study methods
- inclusion and exclusion criteria
- MRI settings used
- founding sources
- declarations of interests.

### 2.7 Study risk of bias assessment

AV and MM assessed the risk of bias of each included study. We resolved any disagreements by consensus.

We used a tailored version of the Quality in Prognosis Studies (QUIPS) tool,^23^ presented in (Appendix S2). Our tailored version of the tool consisted of six domains: study participation, study attrition, prognostic factor measurement, outcome measurement, study confounding, and statistical analysis and reporting.

### 2.8 Summary measures

The summary measure used for our review was Spearman's rank correlation coefficient (r).

### 2.9 Synthesis methods

We used R version 4 “meta” package^24^ and “rob.summary” package^25^ as the software for our data synthesis.

#### 2.9.1 Eligibility for synthesis

All studies that met our eligibility criteria and reported our outcome of interest were assessed to be eligible for quantitative synthesis.

#### 2.9.2 Preparing for synthesis

Given that our effect measure of interest should have been reported directly in the included studies, no data transformation or conversion was deemed necessary. The only exception was the conversion of Pearson's correlation coefficients to Spearman's, which is described above. Although we did not face any situation to perform this conversion, imputation of missing data was not applicable for our effect measure either.

#### 2.9.3 Tabulation and graphical methods

We planned to present the results of each included study with the 95% confidence interval for the effect measure, in conjunction with the synthesized effect estimate, in a forest plot.

#### 2.9.4 Statistical synthesis methods

We used correlation coefficients (*r*) as our summary measure. Most meta‐analysts do not perform syntheses on the correlation coefficient itself because the variance depends strongly on the correlation. Rather, the correlation is converted to the Fisher's z scale and all analyses are performed using the transformed values. We performed a meta‐analysis on converted the Fisher's z scale values based on the random‐effects model. Finally, we converted back Fisher's z to r for the sake of presentation.

#### 2.9.5 Methods to explore heterogeneity

We expected some heterogeneity between studies because of methodological diversity. We evaluated the range of the effects of the random‐effects meta‐analyses using prediction intervals, χ^2^ statistics, and *I* ^2^ statistics. χ^2^ statistics considered to be interpreted as substantial if either τ^2^ was greater than zero, or there was a *p*‐value < 0.10. *I* ^2^ statistic quantifies inconsistency across studies to assess the impact of heterogeneity on the meta‐analysis^26^ and is interpreted as:

- 0%–40%: might not be important;
- 30%–60%: moderate;
- 50%–90%: substantial;
- 75%–100%: considerable.

To investigate the possible sources of heterogeneity, we performed subgroup analyses based on classification of MS (RRMS or SPMS), the diagnostic criteria used for the diagnosis (Poser or McDonald), and the static magnetic field (SMF) used for the MRI (≥1.5T or <1.5T).

#### 2.9.6 Sensitivity analyses

We performed sensitivity analyses on studies with the following factors:

- Component‐based analyses for studies with at least one domain at high or unclear risk of bias
- Very large studies to establish the extent to which they dominate the results.

### 2.10 Reporting bias assessment

To evaluate the risk of reporting bias across studies, a contour‐enhanced funnel plot was generated using Fisher's z transformed correlation for visual inspection of potential publication bias. This plot was designed to have contour lines corresponding to perceived milestones of statistical significance (*p*‐value = 0.01, 0.05, and 0.1). A test for funnel plot asymmetry was conducted.

### 2.11 Certainty assessment

The strength of the overall body of evidence was assessed using an adapted version of the Grading of Recommendations, Assessment, Development and Evaluation (GRADE) framework for prognostic factor research,^27^ which takes into account five considerations: study limitations, inconsistency, indirectness, imprecision, and publication bias. We considered a moderate/large effect size as a criterion for upgrading the certainty of evidence. AV and MM rated the certainty of the evidence for the outcome as “high,” “moderate,” “low,” or “very low”. We resolved any discrepancies by consensus.

## 3 RESULTS

### 3.1 Study selection

#### 3.1.1 Flow of studies

**FIGURE 1:**
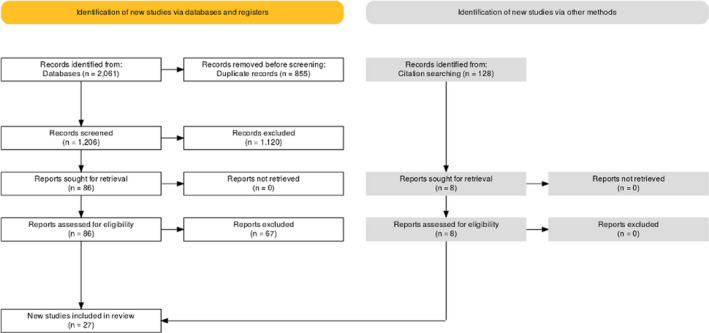
PRISMA Flow diagram of the study

For a detailed summary of the results of the search, see the PRISMA flow diagram presented in Figure 1.

For this review, we identified 2069 records in our primary search (65 CENTRAL, 1192 Embase, 254 MEDLINE through PubMed, and 550 Web of Science). After removing duplicates, we screened the titles and abstracts of 1206 records. Another 1120 records were excluded at that stage, and 86 records remained for full‐text assessment. We excluded 67 studies after assessing the full text of the records. We also found 8 relevant studies through citation searching (Scopus). In the end, we included 27 studies^3^, ^10^, ^11^, ^13^, ^15^, ^16^, ^17^, ^28^, ^29^, ^30^, ^31^, ^32^, ^33^, ^34^, ^35^, ^36^, ^37^, ^38^, ^39^, ^40^, ^41^, ^42^, ^43^, ^44^, ^45^, ^46^, ^47^ for quantitative and qualitative analysis.

#### 3.1.2 Excluded studies

We excluded 59 studies in the full‐text assessment phase. For a full description of the reasons for the exclusion of these 59 studies, see (Appendix S3).

### 3.2 Study characteristics

We included 27 studies with 1919 participants. For a detailed summary of the characteristics of the included studies, see (Appendix S4).

### 3.3 Risk of bias in studies

**FIGURE 2:**
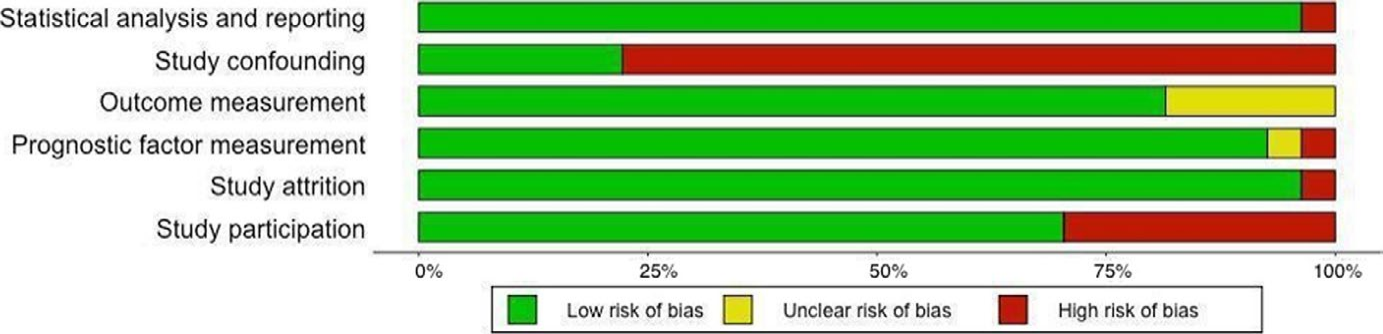
Risk of bias graph: review authors’ judgments about each risk of bias item presented as percentages across all included studies

**FIGURE 3:**
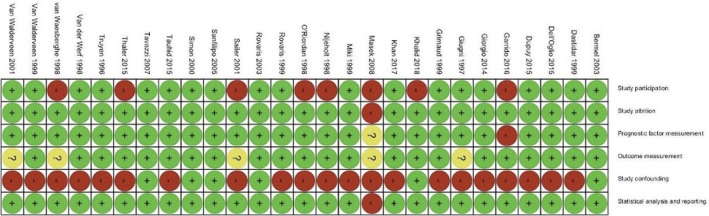
Risk of bias summary: review authors’ judgments about each risk of bias item for each included study

Figure 2 shows the risk of bias judgments for each domain in all included studies. Judgments for each domain across studies are shown in Figure 3.

#### 3.3.1 Study participation

Eight studies were at high risk of bias for this domain,^3^, ^10^, ^15^, ^16^, ^34^, ^37^, ^40^, ^43^ mostly due to inadequate information about the diagnostic criteria used for the diagnosis of MS. The only exception was the study of Giugni et al.^13^ that although did not report the diagnostic criteria used, due to adequate description of participants, we judged that it did not put the study at a higher risk of bias.

#### 3.3.2 Study attrition

Only one study was judged to be at a considerable risk of bias in this domain. Masek et al.^15^ did not provide any information about the patients that were lost to follow‐up or the reason for the loss.

#### 3.3.3 Prognostic factor measurement

Two studies were at risk of bias for this domain. Garrido et al.^43^ did not provide any information about the prognostic factor measurement techniques used and thus was at high risk of bias, while Masek et al.^15^ provided little information regarding this matter for the review authors to be able to judge it and thus was at unclear risk of bias.

#### 3.3.4 Outcome measurement

Five studies were judged to be at unclear risk of bias in this domain^10^, ^13^, ^15^, ^29^, ^34^ because they did not report if the outcome assessors were trained physicians and if the same outcome assessors assessed all the participants.

#### 3.3.5 Study confounding

Most of the included studies were at high risk of bias regarding this domain, due to not reporting or inadequate reporting of the potential confounding factors in their studies. Six studies^17^, ^31^, ^33^, ^36^, ^40^, ^47^ reported potential confounders or the ways used to account for the confounders and thus were judged to be at low risk of bias in this domain.

#### 3.3.6 Statistical analysis and reporting

Twenty‐six studies reported the appropriate effect size (Spearman's rank correlation coefficient) directly. The only exception was the study of Masek et al.^15^ which did not provide enough statistical information about the participants and we had to impute the effect size based on a presented graph.

### 3.4 Results of individual studies

**FIGURE 4:**
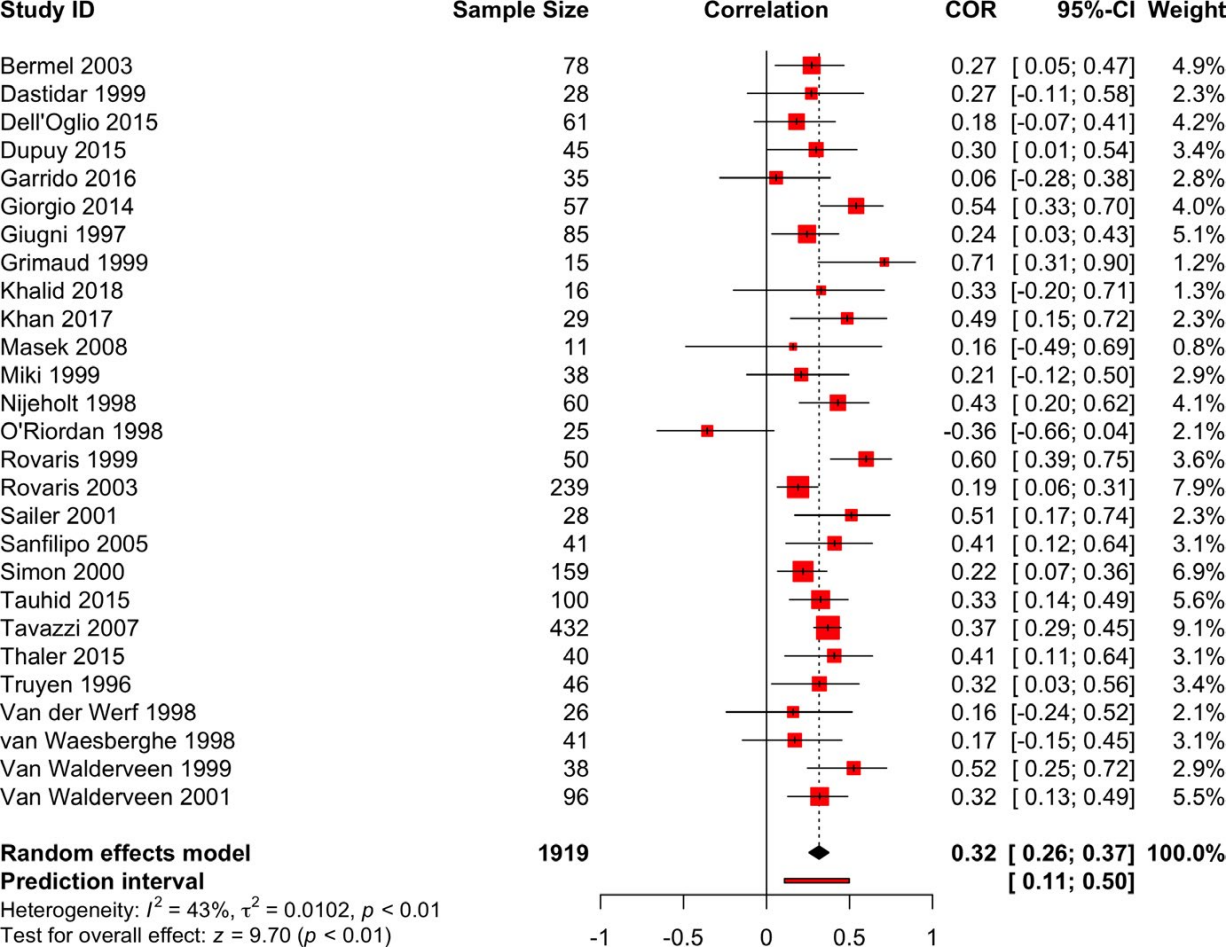
Forest plot of the overall synthesis: The Spearman's rank correlation coefficient (COR) between cerebral MRI T1 hypointense lesion load and EDSS score of RRMS and SPMS participants

All studies reported a positive correlation coefficient, with the only exception being the study of O'Riordan et al.^16^ which reported a negative correlation. In most of the studies, the correlation was also statistically significant. Nine studies concluded that the correlation was not statistically significant.^10^, ^15^, ^16^, ^30^, ^38^, ^40^, ^43^, ^45^, ^46^ For a detailed summary of the results of individual studies, see the forest plot in Figure 4.

### 3.5 Results of syntheses

#### 3.5.1 Characteristics of contributing studies

A summary of the characteristics of the contributing studies is provided in Table 1.

**TABLE 1:** Summary of the characteristics of the contributing studies

Study ID	Sample size			MRI Parameters			Age (SD)
	RRMS	SPMS	Combined	SMF	Sequence	Slice thickness	
Bermel 2003	60	18	78	1.5T	FSE	5 mm	42.5 (8.3)
Dastidar 1999	0	28	0	0.5T	FSE	–	46.0
Dell’Oglio 2015	51	5	61^a^	3.0T	3D MDEFT	1.6 mm	41.0 (8.6)
Dupuy 2015	37	8	45	1.5T	CSE	5 mm	42.3 (8.38)
Garrido 2016	35	0	41^a^	–	–	–	31.2
Giorgio 2014	57	0	57	1.5T	CSE	3 mm	33.8 (8.1)
Giugni 1997	54	31	85	0.5T	CSE	5 mm	33.3
Grimaud 1999	9	6	15	1.5T	CSE & FSE	5 mm	36.0
Khalid 2018	15	0	16^a^	1.5T	CSE	3 mm	33.0 (4.1)
Khan 2017	–	–	39	3.0T	CSE or MP‐RAGE	3 mm	56.2 (6.4)
Masek 2008	0	12	12	–	CSE	–	45.25
Miki 1999	26	12	38	1.5T	FSE	3 mm	43.3 (8.2)
Nijeholt 1998	28	32	60	1.0T	CSE	5 mm	40.9
O'Riordan 1998	0	25	25	1.5T	FSE	5 mm	40.0
Rovaris 1999	32	18	50	1.5T	CSE	5 mm	38.0
Rovaris 2003	239	0	239	Multiple	CSE	3 mm	34.0 (7.5)
Sailer 2001	13	16	29	1.5T	CSE	5 mm	38.2 (6.6)
Sanfilipo 2005	35	6	41	1.5T	CSE	5 mm	39.8 (6.6)
Simon 2000	160	0	160	Multiple	CSE	5 mm	36.3 (6.9)
Tauhid 2015	76	12	100	1.5T	CSE	3 mm	45.5 (9.7)
Tavazzi 2007	294	123	432^a^	1.5T	CSE	5 mm	44.4 (10.2)
Thaler 2015	37	2	40^a^	3.0T	MP‐RAGE	1 mm	36.9 (10.6)
Truyen 1996	29	17	46	0.6T	CSE	5 mm	35.0
Van der Werf 1998	26	19	45	1.0T	CSE	5 mm	37.6 (8.4)
van Waesberghe 1998	8	33	41	1.5T	CSE	5 mm	18–53^b^
Van Walderveen 1999	26	12	38	0.6T	CSE	5 mm	33.0
Van Walderveen 2001	52	44	96	1.0T	CSE	5 mm	39.2

Abbreviations: 3D MDEFT, 3‐Dimensional Modified Driven Equilibrium Fourier Transform; CSE, Conventional Spin Echo; FSE, Fast Spin Echo; MP‐RANGE, Prepared‐Rapid Gradient Echo; RRMS, Relapsing‐Remitting Multiple Sclerosis; SMF, Static Magnetic Field; SPMS, Secondary‐Progressive Multiple Sclerosis.

^a^ PPMS and CIS patients are also included (but less than 15% of the sample size).

^b^ Age of participants is only reported as a range.

##### Design

Fifteen of the included studies were in cross‐sectional design,^3^, ^10^, ^13^, ^16^, ^29^, ^30^, ^31^, ^33^, ^35^, ^37^, ^41^, ^44^, ^45^, ^46^, ^47^ while eleven were cohorts.^11^, ^15^, ^28^, ^32^, ^34^, ^36^, ^38^, ^39^, ^40^, ^42^, ^43^ There was also one trial in our included studies^17^ which we treated like cross‐sectional studies for data extraction and bias assessment.

##### Sample sizes

The median sample size was 41 participants (interquartile range 28.5–69.5). The smallest sample size was 11^15^ and the largest sample size was 432.^31^

##### Setting

Eleven studies were conducted in the USA,^17^, ^31^, ^32^, ^33^, ^38^, ^39^, ^40^, ^44^, ^45^, ^46^, ^47^ four studies were conducted in Netherlands,^28^, ^29^, ^30^, ^37^ three studies were conducted in Italy,^13^, ^35^, ^42^ three studies were conducted in the United Kingdom,^16^, ^34^, ^41^ two studies were conducted in Germany,^3^, ^11^ one study was conducted in Spain,^43^ one study was conducted in Czech Republic,^15^ and two studies were conducted in more than one country (one in Italy and Netherlands^10^; the other in Canada; and multiple sites across Europe^36^).

##### Participants

All studies were conducted on adults. Most studies presented a detailed summary report of the age and sex of the participants. Only two studies^36^, ^41^ did not provide such a detailed summary report.

##### Diagnostic criteria

Eleven studies used the Poser criteria for the diagnosis of MS.^11^, ^17^, ^28^, ^29^, ^30^, ^35^, ^36^, ^38^, ^39^, ^41^, ^46^ Seven studies used one of the variants of the McDonald criteria for this purpose.^31^, ^33^, ^40^, ^42^, ^44^, ^45^, ^47^ Nine studies did not specify which sets of criteria were used for the sake of diagnosis.^3^, ^10^, ^13^, ^15^, ^16^, ^32^, ^34^, ^37^, ^43^

##### MRI parameters

Static magnetic fields, sequences used, and thickness of slices are mentioned here. For a more in‐depth evaluation of the MRI parameters of the included studies, see (Appendix S3).

###### Static magnetic field

Three studies used a static magnetic field (SMF) of 3.0 Tesla,^3^, ^39^, ^45^ thirteen studies used an SMF of 1.5 Tesla,^10^, ^16^, ^31^, ^32^, ^33^, ^34^, ^35^, ^38^, ^40^, ^41^, ^42^, ^44^, ^47^ three studies used 1.0 Tesla,^29^, ^30^, ^37^ two studies used 0.6 T,^11^, ^28^ and two studies used 0.5 T.^13^, ^46^ Two studies did not report their SMF,^15^, ^43^ while two studies used a variety of SMFs.^17^, ^36^

###### Sequence

Most studies used Conventional Spin Echo (CSE) as the MRI sequence. Four studies used Fast Spin Echo (FSE) sequence.^16^, ^38^, ^46^, ^47^ One study used both CSE and FSE.^41^ One study used 3‐Dimensional Modified Driven Equilibrium Fourier Transform (3D MDEFT) sequence.^45^ One study used Magnetization Prepared‐Rapid Gradient Echo (MP‐RAGE).^3^ One multi‐centric study used either CSE or MP‐RAGE.^39^ Finally, one study did not report the sequence used.^43^

###### Slice thickness

Most studies used a slice thickness of 5mm. One study sets slice thickness to 1.6mm (3D MDEFT sequence).^45^ Slice thickness was 1mm in another study (MP‐RAGE sequence).^3^ Six studies used a slice thickness of 3mm.^32^, ^36^, ^38^, ^39^, ^40^, ^42^ Finally, three studies did not report this parameter.^15^, ^43^, ^46^

#### 3.5.2 Results of statistical syntheses

Twenty‐seven studies obtained data sufficient for quantitative synthesis. Results of the meta‐analysis are presented in Figure 4.

A positive r corresponds to a higher EDSS score in participants with larger lesion loads and vice versa.

The pooled sample size was 1919. The pooled estimated Spearman's r was 0.32, with a 95% CI of 0.26–0.37 and a *p*‐value of <0.0001. Thus, we conclude that there is a significant positive correlation between the T1 lesion load and EDSS score. As a rule of thumb, this r is judged to represent a weak to slightly moderate correlation.

#### 3.5.3 Results of investigations of heterogeneity

The study of O’Riordan et al.^16^ appeared to be an outlier, which can be a reason for statistical heterogeneity. The prediction intervals for the summary effect were 0.11–0.50. τ^2^ was 0.0102 with a *p*‐value < 0.01. *I* ^2^ was 43% which is considered as a moderate size of heterogeneity.

For subgroup analysis based on the MS, we had 8 studies^3^, ^11^, ^13^, ^17^, ^36^, ^38^, ^42^, ^43^ in the RRMS subgroup and 6 studies in the SPMS subgroup.^11^, ^13^, ^15^, ^16^, ^38^, ^46^ This synthesis indicated there was no clear difference between groups (test for subgroup differences: χ^2^ = 0.58, df = 1, *p* = 0.44).

For subgroup analysis based on the diagnostic criteria used for the diagnosis, we had 7 studies^31^, ^33^, ^40^, ^42^, ^44^, ^45^, ^47^ in McDonald subgroup 11 studies in the Poser subgroup.^11^, ^17^, ^28^, ^29^, ^30^, ^35^, ^36^, ^38^, ^39^, ^41^, ^46^ Again, there was no difference between the groups (test for subgroup differences: χ^2^ = 0.14, df = 1, *p* = 0.70).

For subgroup analysis based on the SMF used for MRI, only 7 studies had an SMF less than 1.5T.^3^, ^11^, ^13^, ^28^, ^29^, ^37^, ^46^ Statistical synthesis revealed there was no difference between the groups (test for subgroup differences: χ^2^ = 0.29, df =1, *p* = 0.59).

The forest plots for the subgroup analyses are presented in (Appendix S5).

#### 3.5.4 Results of sensitivity analyses

For sensitivity analysis on studies with a large sample size (<90), there were 5 studies.^17^, ^29^, ^31^, ^32^, ^36^ The results of this synthesis were not much different from our overall results (*n* = 1026, *r* = 0.29, 95% CI 0.22–0.36).

We also performed component‐based analysis on the bias domains. Eight studies were at risk of bias for the participation domain.^3^, ^10^, ^16^, ^34^, ^37^, ^40^, ^43^ The results of synthesis on these studies indicated the direction of r was not different in these studies (*n* = 256, *r* = 0.24, 95% CI 0.05–0.41).

Five studies were at risk of bias in the outcome measurement domain.^10^, ^13^, ^15^, ^29^, ^34^ The results of synthesis on these studies indicated the direction of r was not different in these studies (*n* = 261, *r* = 0.29, 95% CI 0.17–0.40).

Twenty‐one studies were at risk of bias in the study confounding domain. The results of synthesis on these studies indicated the direction of r was not different in these studies (*n* = 954, *r* = 0.33, 95% CI 0.25–0.41).

The forest plots for all sensitivity analyses are presented in (Appendix S5). These analyses underline the robustness of the results.

### 3.6 Reporting biases

**FIGURE 5:**
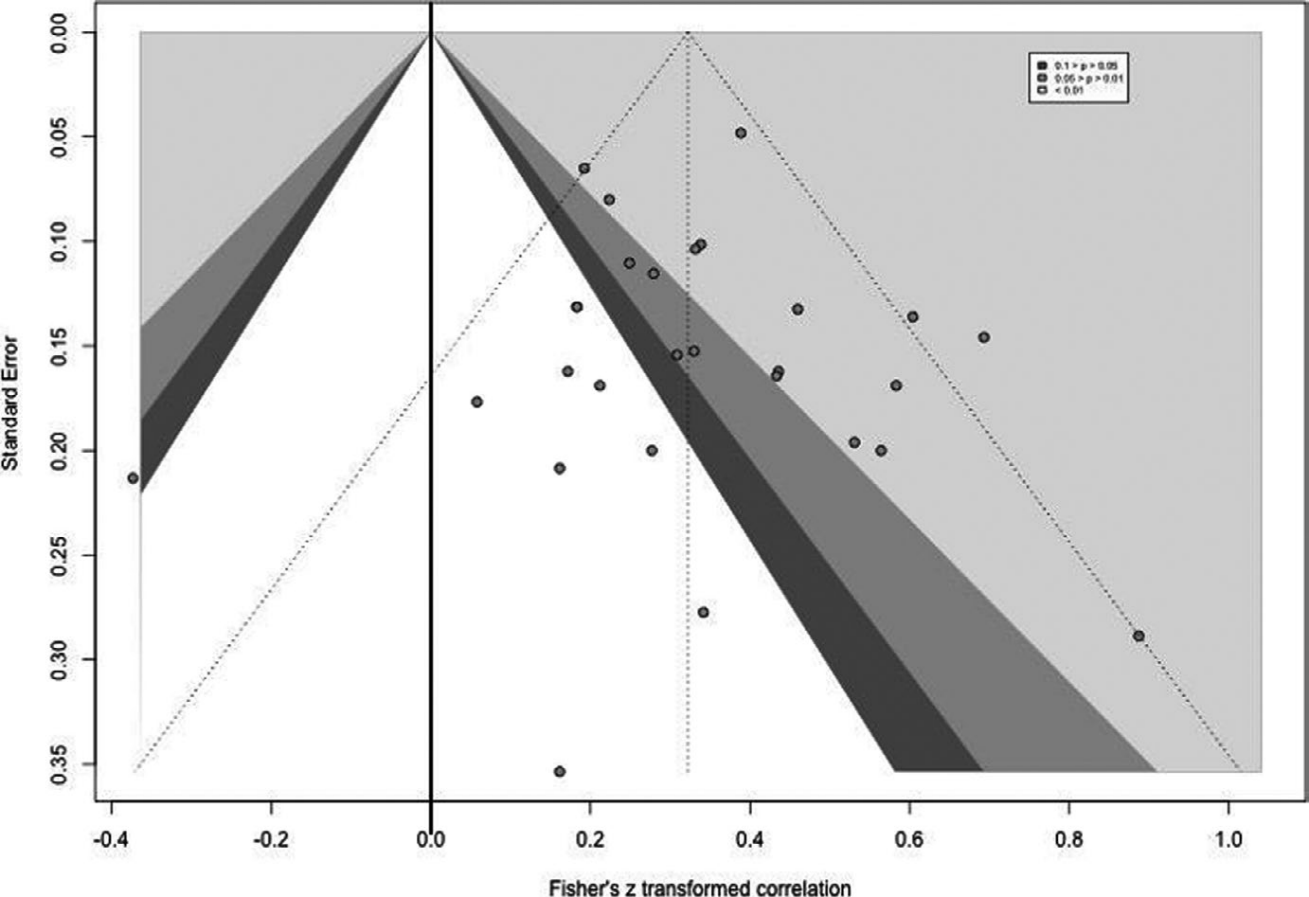
Contour‐enhanced funnel plot

The contour‐enhanced funnel plot is presented in Figure 5.

Visual inspection confirmed that the plot was symmetrical, indicating a low risk for publication bias. Also, a Rank correlation test for funnel plot asymmetry^48^ was performed which yielded non‐significant results (*p* = 0.83), confirming there is no significant risk of publication bias in our review.

### 3.7 Certainty of evidence

#### 3.7.1 Study limitations

Twenty‐two studies were at risk of bias for at least one domain. Five studies were at risk of bias for two domains. Three studies were at risk of bias in three domains. One study was at risk of bias in all six domains. The most common sources of potential bias in these studies were as follows: not appropriately accounting for potential confounders in the design and the analysis, not presenting sufficient data to assess the validity of the diagnosis of participants, and not presenting enough data for validating the outcome measurement methods used. Moreover, we sometimes observed no description of inclusion/exclusion criteria. In general, we considered the included studies to have some serious study limitations. Thus, we decided to downgrade the certainty of evidence by one level.

#### 3.7.2 Inconsistency

The *I* ^2^ was 43% while τ^2^ was 0.0102 with a *p* of less than 0.01. These results indicated moderate inconsistencies in our included studies. Subgroup and sensitivity analyses revealed no difference in the direction of effect in various subsets of studies. Therefore, we decided not to downgrade the certainty of evidence of the results because of this domain.

#### 3.7.3 Indirectness

##### Indirectness in population

In this review, we were interested in adult patients with a diagnosis of either RRMS or SPMS. All of our included studies evaluated this population, and we did not encounter any serious indirectness in this domain.

##### Indirectness in prognostic factor

Our prognostic factor of interest was the cerebral MRI T1 hypointense lesions volume. Considering the specificity of our prognostic factor, there is no indirect way of measuring it. Thus, there are no considerable issues in this domain.

##### Indirectness in outcome

The outcome of interest for our study was the disability measure using EDSS. All included studies used this scale, and thus, there was no issue in this domain either.

#### 3.7.4 Imprecision

The pooled sample size for our review was 1919 which is considered an adequate sample size. 95% CI for the pooled effect size in our meta‐analysis was 0.26–0.37, which represents a weak to slightly moderate correlation. Also, it is worth mentioning that the pooled 95% CI did not overlap the value of no effect. In conclusion, there was no reason for downgrading the certainty of evidence regarding this domain.

#### 3.7.5 Publication bias

We evaluated the risk of the publication bias using a contour‐enhanced funnel plot, observational assessment of the funnel plot for asymmetry, and statistical test of the symmetry of the funnel plot. All of our investigations indicated that there was a low risk for publication bias, so we decided not to downgrade the quality of this evidence for publication bias.

#### 3.7.6 Moderate/large effect size

Our synthesis reported a weak to slightly moderate effect size for the correlation between MRI T1 hypointense lesion load and EDSS score. So, we decided not to upgrade the quality of evidence for the moderate/large effect size domain.

#### 3.7.7 Overall assessment of the confidence in cumulative evidence

We represent the results of the assessments of each domain evaluated for the confidence in cumulative evidence, with overall confidence in cumulative evidence level in Table 2.

**TABLE 2:** Confidence in cumulative evidence

Outcome: Disability measure using Expanded Disability Status Scale (EDSS)									
Prognostic factor	Number of participants	Number of studies	GRADE factors						
			Study limitations	Inconsistency	Imprecision	Publication bias	Moderate/large effect size	Dose effect	Overall quality
Cerebral MRI T1 hypointense lesion load	1919	27	✗	✓	✓	✓	✗	NA	⨁⨁⨁◯

For GRADE factors: ✓, no serious limitations; ✗, serious limitations (or not present for moderate/large effect size, dose‐effect); NA, not applicable. For the overall quality of evidence: ⨁◯◯◯, very low; ⨁⨁◯◯, low; ⨁⨁⨁◯, moderate; ⨁⨁⨁⨁, high.

## 4 DISCUSSION

### 4.1 Interpretation

A variety of quantitative measures derived from conventional and advanced MRI methods have been suggested as prognostic biomarkers for MS. However, as the correlations between different MRI indicators and EDSS have been variable,^49^ no single MRI‐derived measure has been used as a comprehensive prognostic imaging biomarker for MS.^50^ Due to the lack of T2‐hyperintense lesions specificity, attention has been drawn to T1 hypointense lesions, which represent areas of chronic axonal destruction that is more closely linked to current impairment.^5^ Unfortunately, this marker has infrequently been considered, resulting in insufficient evidence on the prognostic impact of this MRI marker. To our knowledge, this is the first systematic review and meta‐analysis that evaluates the value of T1 hypointense lesions’ mean volume as a prognostic factor of disability levels in patients with RRMS or SPMS. Our meta‐analysis of 27 studies showed a weak to a slightly moderate positive correlation between the prognostic factor and the outcome. Subgroup analyses revealed the effect is no different for RRMS or SPMS patients. They also revealed that the diagnostic criteria used for the diagnosis of MS do not affect the magnitude or direction of the correlation. Also, we found that the SMF of the MRI devices used did not result in considerable heterogeneity. Sensitivity analyses revealed that studies with a large sample size or studies at risk of bias in the evaluated domains did not affect the magnitude or direction of the correlation. These analyses underline the robustness of the results. We judged the certainty of the evidence to be high.

Making composites based on a combination of MRI measurements has been suggested in the literature as a promising way to assess the disease's clinical course.^51^ In a study by Poonawalla et al.,^51^ a variety of composites were tested and the Z3d composition was found to be the most powerful: *Z*_nCSF_ (Ratio of CSF volume to total intracranial volume) + *Z*_BH_ (“Black hole”: T1 hypointense lesion volume) + *Z*_T2‐BH_ (Mean T2 value of T1‐lesions). Although it is necessary to mention that each of these measurements necessitates additional imaging sequences, so their practicality remains an area of discussion. Also, since the z‐score varies from person to person, its correlation with EDSS may not be clinically interpretable or practical.^51^

It is also worth mentioning that even though EDSS is commonly used to assess clinical impairment in MS, there is widespread consensus that it lacks sensitivity, and National MS Society Clinical Outcomes Assessment Task Force adopted the Multiple Sclerosis Functional Composite (MSFC) score as an alternative to EDSS.^52^ Future studies can evaluate the correlation between MSFC and MRI measures to obtain more sensitive results.

### 4.2 Limitations of evidence

A large proportion of the included studies were found to be at risk of bias in at least one domain. This factor lowers the certainty of the evidence. Besides that, there were no other serious limitations in our review.

### 4.3 Limitations of review processes

We encountered a considerable number of studies that probably evaluated the prognostic factor and outcome related to our review, but unfortunately, did not report the results. We tried to reach the authors for the data, but were not successful or were not provided with the data. Including those studies could have a substantial effect on our results. Also, a large proportion of the included studies did not mention or account for the potential confounding factors which resulted in downgrading the confidence in our results.

### 4.4 Implications

Our results indicate that the cerebral MRI T1 hypointense lesions load is a weak to moderate prognostic factor in practice to estimate the disability rate of RRMS and SPMS patients. Future studies should evaluate other imaging markers’ validity for this purpose, such as cerebral MRI T2 lesions load or T1 to T2 ratio.

## PROTOCOL

The protocol is published elsewhere.^53^

## AMENDMENTS

As a post hoc decision, we decided to assess the heterogeneity of the included studies by also using *I* ^2^ and τ^2^. We also encountered some studies that reported our outcome of interest at several time points for the same participants. So as another post hoc decision, we decided to average the results of different time points using the formula provided in the methods section. We also found out a large proportion of the studies which reported our prognostic factor and outcome of desire, used the Poser criteria instead of the McDonald criteria for the diagnosis of patients. Thus, we decided to add the Poser criteria as another acceptable method in the population section of our eligibility criteria. We also performed subgroup analyses based on the diagnostic criteria used in the primary studies (Poser or McDonald) and based on the SMF of the MRI in the primary studies (≥1.5T or <1.5T). The rest of the review was done according to our published protocol's methods.

## CONFLICTS OF INTERESTS

None declared.

## AUTHOR CONTRIBUTIONS

AV involved in coordination of the review, designing review, designing the protocol, performing the search, study selection, data extraction, assessing the risk of bias in included studies, analysis of data, interpretation of the results, assessing the confidence in cumulative evidence, and writing the review. EB involved in analysis of data, interpretation of the results, and writing the review. MS involved in conception of the review, designing review, designing the protocol, and writing the review. FA involved in interpretation of the results and writing the review. MF involved in conception of the review, designing review, designing the protocol, and writing the review. MM involved in correspondent, coordination of the review, designing review, designing the protocol, study selection, data extraction, assessing the risk of bias in included studies, assessing the confidence in cumulative evidence, and writing the review.

## Supporting information

Supplementary Material S1

Supplementary Material S2

Supplementary Material S3

Supplementary Material S4

Supplementary Material S5

Supplementary Material S6

Supplementary Material S7

## Data Availability

All the data that were used in the conduction of this review are publicly available as supplementary files.
