# Supplementary material for: Correlation between the clinical disability and T1 hypointense lesions’ volume in cerebral magnetic resonance imaging of multiple sclerosis patients: A systematic review and meta‐analysis
Source: CNS Neurosci Ther. 2021 Oct 3;27(11):1268–80. doi: 10.1111/cns.13734 (PMC8504532; doi:10.1111/cns.13734)
Supplement: Supplementary file 1 — Supplementary Material S1 [file CNS-27-1268-s001.docx]

**Supplementary file A**

**Search strategies**

| **Database and date** | **Search algorithm** |
| --- | --- |
| **Embase**  Elsevier  <1994 to 10.02.2021> | #1 'multiple sclerosis'/exp  #2 (t1 NEAR/5 lesion*):ab,ti,kw  #3 (t1 NEAR/5 hypointens*):ab,ti,kw  #4 'black hole*':ab,ti,kw  #5 #2 OR #3 OR #4  #6 disability:ab,ti,kw  #7 #6 AND #5  #8 #7 AND #1 |
| **MEDLINE**  PubMed  <1946 to 10.02.2021> | Multiple Sclerosis[MeSH Terms] AND ((disability[tiab]) AND ("T1 hypointens*"[tiab] OR "T1 lesion*"[tiab] OR "black hole*"[tiab])) |
| **The Cochrane Library**  10.02.2021 | #1 MeSH descriptor: [Multiple Sclerosis, Relapsing-Remitting] explode all trees  #2 MeSH descriptor: [Multiple Sclerosis, Chronic Progressive] explode all trees  #3 #1 OR #2  #4 (t1 NEAR/5 lesion*):ab,ti,kw  #5 (t1 NEAR/5 hypointens*):ab,ti,kw  #6 'black hole*':ab,ti,kw  #7 #4 OR #5 OR #6  #8 disability:ab,ti,kw  #9 #8 AND #7 |
| **Web of Science**  Science Citation Index – Expanded, Conference Proceedings Citation Index – Science  10.02.2021 | #1 TS="Multiple Sclerosis"  #2 TS=(t1 NEAR/5 lesion*)  #3 TS=(t1 NEAR/5 hypointens*)  #4 TS="black hole*"  #5 #2 OR #3 OR #4  #6 TS="disability"  #7 #6 AND #5  #8 #7 AND #1 |
