# Supplementary material for: Correlation between the clinical disability and T1 hypointense lesions’ volume in cerebral magnetic resonance imaging of multiple sclerosis patients: A systematic review and meta‐analysis
Source: CNS Neurosci Ther. 2021 Oct 3;27(11):1268–80. doi: 10.1111/cns.13734 (PMC8504532; doi:10.1111/cns.13734)
Supplement: Supplementary file 2 — Supplementary Material S2 [file CNS-27-1268-s005.docx]

**Supplementary file B**

**Tailored version of the Quality in Prognosis Studies (QUIPS)[1] tool**

| **Signaling question** | **Authors’ judgment for ‘yes’** |
| --- | --- |
| **Domain 1; Study participation:** | |
| **a. Adequate participation in the study by eligible individuals** | NA: usually participants with information on T1 hypointensity in brain MRI and information on disability levels or relapse rate are selected from a greater study cohort |
| **b. Adequate description of the source population or population of interest** | The source population for the cohort with T1 hypointensity in MRI is clearly described |
| **c. Adequate description of the baseline study sample** | Age and number of participants at baseline is clearly described |
| **d. Adequate description of the sampling frame and recruitment** | Way of establishing the source population, selection criteria, and key characteristics of the source population clearly described |
| **e. Adequate description of the period and place of recruitment** | The period and place of recruitment for both baseline and follow-up visits are clearly described |
| **f. Adequate description of inclusion and exclusion criteria** | Criteria used for diagnosis and classification and description of other inclusion and exclusion criteria are clearly described |
| **Study participation: risk of bias rating (high/low/unclear)** | **High: most items are answered with ’no’; Low: all items answered with ’yes’; Unclear: most items are answered with ’unsure’ Note: potentially a single item may introduce a high risk of bias, depending on study specifics** |
| **Domain 2; Study attrition:** | |
| **a. Adequate response rate for study participants** | NA: usually participants with information on T1 hypointensity in brain MRI and information on disability levels or relapse rate are selected from a greater study cohort |
| **b. Attempts to collect information on participants who dropped out described** | Attempts to collect information on participants who dropped out are described (e.g., telephone contact, mail, registers) |
| **c. Reasons for loss to follow-up provided** | Reasons on participants who dropped out are available (e.g., deceased participants between baseline and follow-up, participants moving to another location) |
| **d. Adequate description of participants lost to follow-up** | Key characteristics of participants lost to follow-up are described (T1 hypointensity load) |
| **e. No important differences between participants who completed the study and those who did not** | Study authors described differences between participants completing the study and those who did not as not important or information provided to judge the differences |
| **Study attrition: risk of bias rating (high/low/unclear)** | **High: most items are answered with ’no’; Low: all items answered with ’yes’; Unclear: most items are answered with ’unsure’ Note: potentially a single item may introduce a high risk of bias, depending on study specifics** |
| **Domain 3; Prognostic factor measurement:** | |
| **a. Clear definition or description provided** | Study authors clearly indicate how they measure T1 hypointense lesions |
| **b. An adequately valid and reliable method of measurement** | NA: cerebral MRI is the only available method for measuring T1 hypointense lesions |
| **c. Continuous variables reported or appropriate cut-points used** | The exact size of the T1 hypointense lesion is reported and it is not categorized base on cut points |
| **d. Same method and setting of measurement used in all study participants** | NA: cerebral MRI is the only available method for measuring T1 hypointense lesions |
| **e. An adequate proportion of the study sample had complete data** | NA: usually participants with information on T1 hypointensity in cerebral MRI and information on disability levels or relapse rate are selected from a greater study cohort |
| **f. Appropriate methods of imputation were used for missing data** | NA: missing radiological size of T1 hypointense lesion can’t be imputed |
| **Prognostic factor measurement: risk of bias rating (high/low/ unclear)** | **High: most items are answered with ’no’; Low: all items answered with ’yes’; Unclear: most items are answered with ’unsure’ Note: potentially a single item may introduce a high risk of bias, depending on study specifics** |
| **Domain 4; Outcome measurement:** | |
| **a. Clear definition of the outcome provided** | Measuring disability level using EDSS should be clearly defined |
| **b. Use of an adequately valid and reliable method of outcome measurement** | EDSS should be measured by a trained physician |
| **c. Use of same method and setting of outcome measurement in all study participants** | Measurements of EDSS are the same for all participants |
| **Outcome measurement: risk of bias rating (high/low/unclear)** | **High: most items are answered with ’no’; Low: all items answered with ’yes’; Unclear: most items are answered with ’unsure’ Note: potentially a single item may introduce a high risk of bias, depending on study specifics** |
| **Domain 5; Study confounding:** | |
| **a. Measurement of all important confounders** | Important confounders are: ethnicity, comedications, and comorbidities, socioeconomic status |
| **b. Provision of clear definitions of the important confounders measured** | Measurement of confounders has to be clearly described |
| **c. Adequately valid and reliable measurement of all important confounders** | Measurement of confounders is valid and reliable |
| **d. Use of same method and setting of confounding measurement in all study participants** | Measurements of confounders are the same for all study participants |
| **e. Appropriate imputation methods used for missing confounders (if applicable)** | Strategy to impute missing confounder data is described |
| **f. Important potential confounders were accounted for in the study design** | Methods section of the publication describes the strategy to account for confounders |
| **g. Important potential confounders were accounted for in the analysis** | Important confounders are accounted for in multivariable logistic regression and Cox proportional hazards models |
| **Study confounding measurement: risk of bias rating (high/ low/unclear)** | **High: most items are answered with ’no’; Low: all items answered with ’yes’; Unclear: most items are answered with ’unsure’ Note: potentially a single item may introduce a high risk of bias, depending on study specifics** |
| **Domain 6; Statistical analysis and reporting:** | |
| **a. Sufficient presentation of data to assess the adequacy of the analytic strategy** | Mean or median values, including confidence intervals or standard errors or standard deviations |
| **b. Strategy for model building is appropriate and based on a conceptual framework or model** | NA: we do not anticipate conceptual frameworks or explicit model building strategies for this type of research question (focusing on one prognostic factor only) |
| **c. Statistical model is adequate for the study design** | Mainly correlation coefficients |
| **d. No selective reporting of results** | NA: EDSS score and relapse rate are the only outcomes; if missing the study will be excluded |
| **Statistical analysis and reporting: risk of bias rating (high/ low/unclear)** | **High: most items are answered with ’no’; Low: all items answered with ’yes’; Unclear: most items are answered with ’unsure’ Note: potentially a single item may introduce a high risk of bias, depending on study specifics** |
| **No: no or no relevant information to answer the signaling question**  **Partly: information is provided but not completely**  **Unsure: not enough information to answer signaling question with yes or no**  **NA (not applicable): signaling question not appropriate for this type of prognostic review** | |

# **References**

1. Hayden, J.A., P. Cote, and C. Bombardier, *Evaluation of the quality of prognosis studies in systematic reviews.* Ann Intern Med, 2006. **144**(6): p. 427-37.
