# Supplementary material for: Correlation between the clinical disability and T1 hypointense lesions’ volume in cerebral magnetic resonance imaging of multiple sclerosis patients: A systematic review and meta‐analysis
Source: CNS Neurosci Ther. 2021 Oct 3;27(11):1268–80. doi: 10.1111/cns.13734 (PMC8504532; doi:10.1111/cns.13734)
Supplement: Supplementary file 3 — Supplementary Material S3 [file CNS-27-1268-s007.docx]

# Supplementary file C

## Characteristics of included studies

[Ordered by study ID]

**List of abbreviations**

| **Abbreviation** | **Term** |
| --- | --- |
| 3D MDEFT | 3-dimensional modified driven equilibrium Fourier transform |
| CSE | Conventional spin-echo |
| FOV | Field of view |
| FSE | Fast spin-echo |
| MP-RAGE | Magnetization prepared-rapid gradient-echo |
| RRMS | Relapsing-remitting multiple sclerosis |
| SMF | Static magnetic field |
| SPMS | Secondary-progressive multiple sclerosis |
| TE | Echo time |
| TR | Repetition time |

### Bermel 2003[1]

| Methods | Cross-sectional  Setting: Outpatients  Single-center  Country: USA |
| --- | --- |
| Participants | 78 participants (60 RRMS and 18 SPMS)  Mean age (SD): 42.5 (8.3)  Sex (M:F): 27/51  Inclusion criteria:   1. age 20-60; and 2. established MS diagnosis of either RRMS or SPMS by the McDonald criteria.   Exclusion criteria:   1. patients who experienced acute exacerbations or underwent corticosteroid treatment within the previous 4 weeks before clinical and MRI testing; 2. other major medical illnesses; 3. age less than 20 or greater than 60 years; and 4. history of substance abuse. |
| Prognostic factor | Equipment properties: Scanner: Philips Gyroscan; SMF: 1.5T; Sequence: FSE; TR: 400; TE: 10; Matrix: 192x256; FOV: -; Slices: 24; Thickness: 5mm; Inter-slice gap: 0mm.  Confounding factors: none |
| Notes | Funding source: Supported by an Alpha Omega Alpha summer research grant (R. Bermel), University at Buffalo Summer Research Fellowship (R. Bermel), and National Institute of Health NIH-NINDS 1 K23 NS42379-01 (R. Bakshi).  Conflicts of interest: unknown |

| Bias | Author’s judgment |
| --- | --- |
| Study participation | Low risk |
| Study attrition | Low risk |
| Prognostic factor measurement | Low risk |
| Outcome measurement | Low risk |
| Study confounding | Low risk |
| Statistical analysis and reporting | Low risk |

### Dastidar 1999[2]

| Methods | Cross-sectional  Setting: Inpatients and outpatients  Single-center  Country: USA |
| --- | --- |
| Participants | 28 participants (all SPMS)  Mean age (range): 46.0 (35 – 58)  Sex (M:F): 14/14  Inclusion criteria:   1. definite MS diagnosis by the Poser criteria.   Exclusion criteria:   1. patients who received any kind of steroids; and 2. steroid administration within the last 6 months. |
| Prognostic factor | Equipment properties: Scanner: -; SMF: 0.5T; Sequence: FSE; TR: 300; TE: 20; Matrix: 160x256; FOV: 22cm; Slices: -; Thickness: -; Inter-slice gap: -.  Confounding factors: unknown |
| Notes | Funding source: Medical Research Fund of the Tampere University Hospital  Conflicts of interest: unknown |

| Bias | Author’s judgment |
| --- | --- |
| Study participation | Low risk |
| Study attrition | Low risk |
| Prognostic factor measurement | Low risk |
| Outcome measurement | Low risk |
| Study confounding | High risk |
| Statistical analysis and reporting | Low risk |

### Dell’Oglio 2015[3]

| Methods | Cross-sectional  Setting: Inpatients and outpatients  Single-center  Country: USA |
| --- | --- |
| Participants | 61 participants (4 CIS, 1 PPMS, 51 RRMS, 5 SPMS)  Mean age (SD): 41.0 (8.6)  Sex (M:F): 19/42  Inclusion criteria:   1. age 18-55; and 2. established MS diagnosis of either RRMS, SPMS, PPMS, or CIS by the McDonald criteria.   Exclusion criteria:   1. other major medical, neurologic, or neuropsychiatric disorder; 2. relapse or corticosteroid use in the 4 weeks before MRI (to avoid transient confounding effects on MRI); 3. initiated on disease-modifying therapy in the 6 months preceding MRI (to avoid transient confounding effects on MRI of newly started therapy such as pseudoatrophy); and 4. history of substance abuse or smoking. |
| Prognostic factor | Equipment properties: Scanner: Signa imager; SMF: 3.0T; Sequence: 3D MDEFT; TR: 7.9; TE: 3.14; Matrix: 256x256; FOV: -; Slices: 124; Thickness: 1.6mm; Inter-slice gap: 0mm.  Confounding factors: unknown |
| Notes | Funding source: unknown  Conflicts of interest: unknown |

| Bias | Author’s judgment |
| --- | --- |
| Study participation | Low risk |
| Study attrition | Low risk |
| Prognostic factor measurement | Low risk |
| Outcome measurement | Low risk |
| Study confounding | Low risk |
| Statistical analysis and reporting | Low risk |

### Dupuy 2015[4]

| Methods | Cross-sectional  Setting: Inpatients and outpatients  Single-center  Country: USA |
| --- | --- |
| Participants | 45 participants (37 RRMS and 8 SPMS)  Mean age (SD): 42.3 (8.38)  Sex (M:F): 12/33  Inclusion criteria:   1. established MS diagnosis of either RRMS or SPMS by the McDonald criteria.   Exclusion criteria: - |
| Prognostic factor | Equipment properties: Scanner: Philips Gyroscan; SMF: 1.5T; Sequence: CSE; TR: 400; TE: 10; Matrix: -; FOV: -; Slices: 24; Thickness: 5mm; Inter-slice gap: 0mm.  Confounding factors: unknown |
| Notes | Funding source: unknown  Conflicts of interest: authors declared no conflicts of interest |

| Bias | Author’s judgment |
| --- | --- |
| Study participation | Low risk |
| Study attrition | Low risk |
| Prognostic factor measurement | Low risk |
| Outcome measurement | Low risk |
| Study confounding | High risk |
| Statistical analysis and reporting | Low risk |

### Garrido 2016[5]

| Methods | Prospective cohort  Setting: Inpatients and outpatients  Single-center  Country: Spain  Length of follow-up: 2 years |
| --- | --- |
| Participants | 41 participants (6 CIS and 35 RRMS)  Median age (range): 31.2 (18 – 48)  Sex (M:F): 14/27  Inclusion criteria:   1. untreated RRMS or CIS patients.   Exclusion criteria: - |
| Prognostic factor | Equipment properties: -  Confounding factors: unknown |
| Notes | Funding source: unknown  Conflicts of interest: unknown |

| Bias | Author’s judgment |
| --- | --- |
| Study participation | High risk |
| Study attrition | Low risk |
| Prognostic factor measurement | High risk |
| Outcome measurement | Low risk |
| Study confounding | High risk |
| Statistical analysis and reporting | Low risk |

### Giorgio 2014[6]

| Methods | Prospective cohort  Setting: Inpatients and outpatients  Single-center  Country: Italy |
| --- | --- |
| Participants | 57 participants (all RRMS)  Mean age (SD): 33.8 (8.1)  Sex (M:F): 15/42  Inclusion criteria:   1. established MS diagnosis of RRMS by the McDonald criteria.   Exclusion criteria: - |
| Prognostic factor | Equipment properties: Scanner: Philips Gyroscan; SMF: 1.5T; Sequence: CSE; TR: 35; TE: 10; Matrix: 256x256; FOV: 25cm; Slices: 50; Thickness: 3mm; Inter-slice gap: 0mm.  Confounding factors: unknown |
| Notes | Funding source: This work was supported by a grant from FISM—Fondazione Italiana Sclerosi Multipla—Cod. 2010/R/15.  Conflicts of interest: authors declared no conflicts of interest |

| Bias | Author’s judgment |
| --- | --- |
| Study participation | Low risk |
| Study attrition | Low risk |
| Prognostic factor measurement | Low risk |
| Outcome measurement | Low risk |
| Study confounding | High risk |
| Statistical analysis and reporting | Low risk |

### Giugni 1997[7]

| Methods | Cross-sectional  Setting: unknown  Single-center  Country: Italy |
| --- | --- |
| Participants | 85 participants (54 RRMS and 31 SPMS)  Mean age (SD): 29.7 (7.5) RRMS, 39.6 (7.8) SPMS  Sex (M:F): 27/58  Inclusion criteria:   1. RRMS defined as those with a history of relapses and remissions without gradual deterioration; and 2. those presenting with an initial RR course followed by progressive deterioration for at least 6 months with or without superimposed relapses.   Exclusion criteria:   1. Patients with RR-MS but disease duration > 10 years and EDSS score <3 (benign cases) |
| Prognostic factor | Equipment properties: Scanner: Toshiba 50 S; SMF: 0.5T; Sequence: CSE; TR: 400; TE: 18; Matrix: 256x160; FOV: 25cm; Slices: -; Thickness: 5mm; Inter-slice gap: 1mm.  Confounding factors: none |
| Notes | Funding source: unknown  Conflicts of interest: unknown |

| Bias | Author’s judgment |
| --- | --- |
| Study participation | Low risk |
| Study attrition | Low risk |
| Prognostic factor measurement | Low risk |
| Outcome measurement | Unclear risk |
| Study confounding | High risk |
| Statistical analysis and reporting | Low risk |

### Grimaud 1999[8]

| Methods | Cross-sectional  Setting: Outpatients  Single-center  Country: United Kingdom |
| --- | --- |
| Participants | 15 participants (9 RRMS and 6 SPMS)  Median age (range): 36.0 (29 - 45)  Sex (M:F): unknown  Inclusion criteria:   1. Clinically definite MS based on Poser criteria, and 2. No evidence of other pathology.   Exclusion criteria: - |
| Prognostic factor | Equipment properties: Scanner: Signa imager; SMF: 1.5T; Sequence: CSE and FSE; TR: 400; TE: 18; Matrix: 256x128; FOV: 24cm; Slices: 18; Thickness: 5mm; Inter-slice gap: 2.5mm.  Confounding factors: unknown |
| Notes | Funding source: Funded by a grant from the Multiple Sclerosis Society of Great Britain and Northern Ireland, and supported by the Brain Research Trust.  Conflicts of interest: J.G. was supported by a grant from the Association pour la Recherche sur la Sclérose en Plaques, France, and Lilly Industries, UK. |

| Bias | Author’s judgment |
| --- | --- |
| Study participation | Low risk |
| Study attrition | Low risk |
| Prognostic factor measurement | Low risk |
| Outcome measurement | Low risk |
| Study confounding | High risk |
| Statistical analysis and reporting | Low risk |

### Khalid 2018[9]

| Methods | Retrospective cohort  Setting: Inpatients and outpatients  Single-center  Country: USA  Length of follow-up: 1 year |
| --- | --- |
| Participants | 16 participants (15 RRMS and 1 CIS)  Mean age (SD): 33.0 (4.1)  Sex (M:F): 0/16  Inclusion criteria:   1. established MS diagnosis of MS by the McDonald criteria. 2. Participants should have a brain MRI available within six months before pregnancy and within 6 months after delivery.   Exclusion criteria: - |
| Prognostic factor | Equipment properties: Scanner: Signa imager; SMF: 1.5T; Sequence: CSE; TR: 467-733; TE: 20; Matrix: -; FOV: -; Slices: 54; Thickness: 3mm; Inter-slice gap: 0mm.  Confounding factors: pregnancy was not a confounding factor in this study since the data was collected before pregnancy. |
| Notes | Funding source: unknown  Conflicts of interest: unknown |

| Bias | Author’s judgment |
| --- | --- |
| Study participation | High risk |
| Study attrition | Low risk |
| Prognostic factor measurement | Low risk |
| Outcome measurement | Low risk |
| Study confounding | Low risk |
| Statistical analysis and reporting | Low risk |

### Khan 2017[10]

| Methods | Retrospective cohort  Setting: Outpatients  Multicenter  Country: USA  Length of follow-up: 3 years |
| --- | --- |
| Participants | 39 participants (all MS)  Mean age (SD): 56.2 (6.4)  Sex (M:F): 13/26  Inclusion criteria:   1. Definite MS based on Poser criteria; 2. Have an EDSS score between 0 and 5; and 3. Have at least two relapses in the 2 years before study randomization.   Exclusion criteria: - |
| Prognostic factor | Equipment properties: Scanner: -; SMF: 3.0T; Sequence: CSE or MP-RAGE; TR: 8710-45009; TE: 82-95; Matrix: 256x256; FOV: -; Slices: -; Thickness: 3mm; Inter-slice gap: -.  Confounding factors: Glatiramer Acetate |
| Notes | Funding source: This study was funded by Teva Pharmaceutical Industries, Petach Tikva, Israel. Natalia Ashtamker, Scott Kolodny, and Yulia Sidi are employees of Teva Pharmaceutical Industries.  Conflicts of interest: Omar Khan has received compensation for consulting from Biogen Idec, Genzyme, and Novartis and for serving on speaker bureaus from Teva Pharmaceutical Industries, Novartis, and Biogen, and he has received research support from the NIH, NINDS, NMSS, Teva Pharmaceutical Industries, Biogen, Genzyme, Roche, and Novartis. |

| Bias | Author’s judgment |
| --- | --- |
| Study participation | Low risk |
| Study attrition | Low risk |
| Prognostic factor measurement | Low risk |
| Outcome measurement | Low risk |
| Study confounding | High risk |
| Statistical analysis and reporting | Low risk |

### Masek 2008[11]

| Methods | Prospective cohort  Setting: Outpatients  Single-center  Country: Czech Republic  Length of follow-up: 3 years |
| --- | --- |
| Participants | 12 participants (all SPMS)  Mean age (range): 45.25 (32 - 54)  Sex (M:F): 9/3  Inclusion criteria:   1. established MS diagnosis of SPMS.   Exclusion criteria: - |
| Prognostic factor | Equipment properties: Scanner: -; SMF: 3.0T; Sequence: CSE; TR: 536; TE: 14; Matrix: -; FOV: -; Slices: -; Thickness: -; Inter-slice gap: -.  Confounding factors: unknown |
| Notes | Funding source: Ministry of Health of Czech Republic (MZO/00064165 and MSMOO 21620849)  Conflicts of interest: unknown |

| Bias | Author’s judgment |
| --- | --- |
| Study participation | High risk |
| Study attrition | High risk |
| Prognostic factor measurement | Unclear risk |
| Outcome measurement | Unclear risk |
| Study confounding | High risk |
| Statistical analysis and reporting | High risk |

### Miki 1999[12]

| Methods | Prospective cohort  Setting: Outpatients  Single-center  Country: USA |
| --- | --- |
| Participants | 38 participants (26 RRMS and 12 SPMS)  Mean age (SD): 43.3 (8.2)  Sex (M:F): 13/25  Inclusion criteria:   1. Definite MS based on Poser criteria; 2. Patients with RRMS had had at least two relapses over the preceding 2 years, with a relapse defined as a new neurologic deficit or exacerbation of a previous deficit, confirmed using examination, that developed over 1–5 days when conditions had previously been stable and that lasted at least 48 hours; and 3. Patients with SPMS had an increase in the Expanded Disability Status Scale score of at least 1.0 over the preceding year without an acute exacerbation.   Exclusion criteria:   1. Patients treated with immunosuppressants or cytotoxic or immunomodulatory drugs, other than brief courses of pulsed corticosteroids to treat exacerbations. |
| Prognostic factor | Equipment properties: Scanner: Signa imager; SMF: 1.5T; Sequence: CSE; TR: 600; TE: 11; Matrix: 256x192; FOV: 22cm; Slices: -; Thickness: 3mm; Inter-slice gap: 0mm.  Confounding factors: unknown |
| Notes | Funding source: U.S. National Institutes of Health grants R01 NS2 9029-01A1 and M01- RR00040.  Conflicts of interest: unknown |

| Bias | Author’s judgment |
| --- | --- |
| Study participation | Low risk |
| Study attrition | Low risk |
| Prognostic factor measurement | Low risk |
| Outcome measurement | Low risk |
| Study confounding | High risk |
| Statistical analysis and reporting | Low risk |

### Nijeholt 1998[13]

| Methods | Cross-sectional  Setting: Outpatients  Single-center  Country: Netherlands |
| --- | --- |
| Participants | 60 participants (28 RRMS, 32 SPMS)  Median age (range): 35 (27 – 57) RRMS, 46 (30 – 65) SPMS  Sex (M:F): 37/54  Inclusion criteria:   1. established MS diagnosis of either RRMS, SPMS, or PPMS.   Exclusion criteria: - |
| Prognostic factor | Equipment properties: Scanner: Magnetom Impact; SMF: 1.0T; Sequence: CSE; TR: 600; TE: 15; Matrix: 256x512; FOV: 24cm; Slices: 21; Thickness: 5mm; Inter-slice gap: 0.5mm.  Confounding factors: unknown |
| Notes | Funding source: Dutch Multiple Sclerosis Society (grant 92–131).  Conflicts of interest: unknown |

| Bias | Author’s judgment |
| --- | --- |
| Study participation | High risk |
| Study attrition | Low risk |
| Prognostic factor measurement | Low risk |
| Outcome measurement | Low risk |
| Study confounding | High risk |
| Statistical analysis and reporting | Low risk |

### O'Riordan 1998[14]

| Methods | Cross-sectional  Setting: Outpatients  Single-center  Country: United Kingdom |
| --- | --- |
| Participants | 25 participants (all SPMS)  Mean age: 40.0  Sex (M:F): 11/14  Inclusion criteria:   1. male and female patients; 2. aged between 18 and 50 years; and 3. secondary progressive multiple sclerosis (Patients were considered in the secondary progressive phase if after an initial relapsing-remitting course there followed a progressive deterioration of at least 6 months duration unrelated to exacerbation.   Exclusion criteria: - |
| Prognostic factor | Equipment properties: Scanner: Signa imager; SMF: 1.5T; Sequence: FSE; TR: 600; TE: 14; Matrix: 256x256; FOV: 25cm; Slices: -; Thickness: 5mm; Inter-slice gap: -.  Confounding factors: Campath 1H (anti-CD52 monoclonal antibody). |
| Notes | Funding source: unknown  Conflicts of interest: unknown |

| Bias | Author’s judgment |
| --- | --- |
| Study participation | High risk |
| Study attrition | Low risk |
| Prognostic factor measurement | Low risk |
| Outcome measurement | Low risk |
| Study confounding | High risk |
| Statistical analysis and reporting | Low risk |

### Rovaris 1999[15]

| Methods | Cross-sectional  Setting: Outpatients  Single-center  Country: Italy |
| --- | --- |
| Participants | 50 participants (32 RRMS and 18 SPMS)  Mean age (range): 38.0 (19 – 65)  Sex (M:F): 17/33  Inclusion criteria:   1. definite MS based on Poser criteria; and 2. Diagnosis of RRMS or SPMS.   Exclusion criteria:   1. Patients who were in a clinical relapse phase at the time of study entry. |
| Prognostic factor | Equipment properties: Scanner: -; SMF: 1.5T; Sequence: CSE; TR: 768; TE: 14; Matrix: 256x256; FOV: 25cm; Slices: 24; Thickness: 5mm; Inter-slice gap: 0mm.  Confounding factors: unknown |
| Notes | Funding source: A grant from the Associazione Italiana Sclerosi Multipla (Genova, Italy).  Conflicts of interest: unknown |

| Bias | Author’s judgment |
| --- | --- |
| Study participation | Low risk |
| Study attrition | Low risk |
| Prognostic factor measurement | Low risk |
| Outcome measurement | Low risk |
| Study confounding | High risk |
| Statistical analysis and reporting | Low risk |

### Rovaris 2003[16]

| Methods | Retrospective cohort  Setting: Inpatients and outpatients  Multicenter  Country: Europe and Canada  Length of follow-up: 9m |
| --- | --- |
| Participants | 239 participants (all RRMS)  Mean age (SD): 34.0 (7.5)  Sex (M:F): unknown  Inclusion criteria:   1. age of 18-50 years; 2. definite RRMS based on Poser criteria for at least 1 year; 3. EDSS 0.0-5.0; 4. At least one documented relapse in the preceding 2 years; and 5. At least one contrast-enhancing lesion on their screening brain MR images.   Exclusion criteria:   1. relapse or steroid treatment in the 30 days before their inclusion into the study; 2. prior lymphoid irradiation, the use of immunosuppressant or cytotoxic agents in the past 2 years; 3. use of azathioprine, cyclosporine, interferons, Deoxyspergualine, or chronic corticosteroids during the previous 6 months; 4. subjects receiving concomitant therapy with an experimental drug for MS or another disease; 5. patients with other serious intercurrent systemic or psychiatric illnesses; |
| Prognostic factor | Equipment properties: Scanner: -; SMF: 21 at 1.5T, 5 at 1.0T, and 2 at 0.5T; Sequence: CSE; TR: 450-650; TE: 10-20; Matrix: -; FOV: -; Slices: 44; Thickness: 3mm; Inter-slice gap: 0mm.  Confounding factors: GA, 20 mg daily by subcutaneous injection. |
| Notes | Funding source: Supported by Teva Pharmaceutical, Ltd.  Conflicts of interest: unknown |

| Bias | Author’s judgment |
| --- | --- |
| Study participation | Low risk |
| Study attrition | Low risk |
| Prognostic factor measurement | Low risk |
| Outcome measurement | Low risk |
| Study confounding | Low risk |
| Statistical analysis and reporting | Low risk |

### Sailer 2001[17]

| Methods | Retrospective cohort  Setting: Outpatients  Single-center  Country: United Kingdom  Length of follow-up: 12 months |
| --- | --- |
| Participants | 29 participants (13 RRMS and 16 SPMS)  Mean age (SD): 38.2 (6.6)  Sex (M:F): 13/16  Inclusion criteria:   1. patients with clinically definite multiple sclerosis that followed a relapsing-remitting or secondary progressive course; 2. evidence of clinically active disease (either two relapses within the 12 months before inclusion, one of which was in the preceding 6months or deterioration of at least one point on the EDSS within the preceding 18 months); and 3. a current EDSS score of 2-7.   Exclusion criteria:   1. diagnosis of PPMS; 2. significant cognitive impairment; and 3. treatment with steroids within the last month. |
| Prognostic factor | Equipment properties: Scanner: Signa imager; SMF: 1.5T; Sequence: CSE; TR: 600; TE: 30; Matrix: 256x256; FOV: 24cm; Slices: -; Thickness: 5mm; Inter-slice gap: -.  Confounding factors: anti-CD4 drugs |
| Notes | Funding source: MS Society of Great Britain and Northern Ireland.  Conflicts of interest: unknown |

| Bias | Author’s judgment |
| --- | --- |
| Study participation | High risk |
| Study attrition | Low risk |
| Prognostic factor measurement | Low risk |
| Outcome measurement | Unclear risk |
| Study confounding | High risk |
| Statistical analysis and reporting | Low risk |

### Sanfilipo 2005[18]

| Methods | Cross-sectional  Setting: Inpatients and outpatients  Single-center  Country: USA |
| --- | --- |
| Participants | 41 participants (35 RRMS and 6 SPMS)  Mean age (SD): 39.8 (6.6)  Sex (M:F): 9/32  Inclusion criteria:   1. established MS diagnosis of either RRMS or SPMS by the McDonald criteria.   Exclusion criteria:   1. age less than 21 or greater than 50 years; 2. pregnancy; 3. other major medical illness; 4. past or current substance abuse; 5. corticosteroid use in the previous 4 weeks; and 6. neurologic disorders other than MS. |
| Prognostic factor | Equipment properties: Scanner: Philips Gyroscan; SMF: 1.5T; Sequence: CSE; TR: 400; TE: 10; Matrix: 192x256; FOV: 25cm; Slices: 24; Thickness: 5mm; Inter-slice gap: 0mm.  Confounding factors: none |
| Notes | Funding source: This research was supported in part by an Alpha Omega Alpha Student Research Fellowship (M. Sanfilipo), a University at Buffalo School of Medicine and Biological Sciences Summer Research Fellowship (M. Sanfilipo), and by research grants from the National Institutes of Health (NIH-NINDS 1 K23 NS42379-01, R. Bakshi), National Multiple Sclerosis Society (RG 3258A2/1, BWG, R. Bakshi; RG 3574A1, R. Bakshi), and National Science Foundation (DBI- 0234895, BWG, R. Bakshi).  Conflicts of interest: unknown |

| Bias | Author’s judgment |
| --- | --- |
| Study participation | Low risk |
| Study attrition | Low risk |
| Prognostic factor measurement | Low risk |
| Outcome measurement | Low risk |
| Study confounding | Low risk |
| Statistical analysis and reporting | Low risk |

### Simon 2000[19]

| Methods | Clinical trial  Setting: Inpatients and outpatients  Multicenter  Country: USA  Length of follow-up: 2 years |
| --- | --- |
| Participants | 160 participants (all RRMS)  Mean age (SD): 36.3 (6.9)  Sex (M:F): 35/125  Inclusion criteria:   1. Ages between 18 and 55; 2. definite diagnosis of RRMS of at least 1-year duration (based on Poser criteria) 3. at least two exacerbations in the 3 years before study entry; 4. free of exacerbations for at least 2 months before study entry; and 5. EDSS greater than or equal to 1.0 but less than or equal to 3.5.   Exclusion criteria:   1. have not experienced an exacerbation within the 2 months before study entry; 2. prior therapy with immunosuppressant drugs or interferon; 3. treatment with ACTH or corticosteroids within 2 months before study entry; 4. concurrent infection; 5. the presence of any serious disease, other than MS; 6. chronic progressive MS; and 7. pregnant women or nursing mothers. |
| Prognostic factor | Equipment properties: Scanner: 6 with Philips Gyroscan and 16 with Signa imager; SMF: 14 at 1.5T and 8 at 1.0T; Sequence: CSE; TR: 600; TE: 20; Matrix: 192x256; FOV: 24cm; Slices: -; Thickness: 5mm; Inter-slice gap: 0mm.  Confounding factors: 30 mcg (6 million IU) IFN-beta-1a (Avonex; Biogen, Inc., Cambridge, MA) once weekly by IM injection. |
| Notes | Funding source: National Institutes of Health, NINDS R01-26321, and Biogen, Inc., Cambridge, MA.  Conflicts of interest: unknown |

| Bias | Author’s judgment |
| --- | --- |
| Study participation | Low risk |
| Study attrition | Low risk |
| Prognostic factor measurement | Low risk |
| Outcome measurement | Low risk |
| Study confounding | Low risk |
| Statistical analysis and reporting | Low risk |

### Tauhid 2015[20]

| Methods | Prospective cohort  Setting: Inpatients  Single-center  Country: USA  Length of follow-up: 7 years |
| --- | --- |
| Participants | 100 participants (76 RRMS, 12 SPMS, and 6 PPMS)  Mean age (SD): 45.5 (9.7)  Sex (M:F): 25/75  Inclusion criteria:   1. age ≥18 years; and 2. a definitive diagnosis of MS within the last 3 years.   Exclusion criteria: - |
| Prognostic factor | Equipment properties: Scanner: Signa imager; SMF: 1.5T; Sequence: CSE; TR: 725; TE: 20; Matrix: -; FOV: -; Slices: -; Thickness: 3mm; Inter-slice gap: -.  Confounding factors: unknown |
| Notes | Funding source: This study was funded by Novartis Pharmaceuticals. It is based on data from the Comprehensive Longitudinal Investigation of Multiple Sclerosis at Brigham and Women’s Hospital (CLIMB) study, funded in part by Merck Serono.  Conflicts of interest: Rahul Sasane is an employee of Novartis Pharmaceuticals. Bonnie Glanz and Brian Healy received research support from Merck Serono. Tanuja Chitnis received consulting fees from Biogen, Merck Serono, and Alexion and research support from Merck Serono and Novartis. Howard Weiner received personal compensation from Biogen, Novartis, EMD Serono, Teva, GSK, Nasvax, Xenoport, and Genzyme and research support from Merck Serono. Rohit Bakshi received consulting fees from AbbVie, Alkermes, Biogen, Novartis, and Questcor and research support from Biogen, Merck Serono, Novartis, Genzyme, and Teva. The other authors state that there is no conflict of interest. |

| Bias | Author’s judgment |
| --- | --- |
| Study participation | Low risk |
| Study attrition | Low risk |
| Prognostic factor measurement | Low risk |
| Outcome measurement | Low risk |
| Study confounding | High risk |
| Statistical analysis and reporting | Low risk |

### Tavazzi 2007[21]

| Methods | Cross-sectional  Setting: Inpatients and outpatients  Single-center  Country: USA |
| --- | --- |
| Participants | 432 participants (294 RRMS, 123 SPMS, and 15 PPMS)  Mean age (SD): 44.4 (10.2)  Sex (M:F): 86/346  Inclusion criteria:   1. MRI examination performed at the time of their clinical visit; 2. age 18–70 years; 3. EDSS 0–8.5; and 4. established MS diagnosis of either RRMS or SPMS by the McDonald criteria.   Exclusion criteria:   1. relapse; 2. disease progression and steroid treatment in the 3 months preceding study entry; and 3. pre-existing medical conditions known to be associated with brain pathology (e.g., a neurodegenerative disorder, cerebrovascular disease, positive history of alcohol abuse, etc.). |
| Prognostic factor | Equipment properties: Scanner: Signa imager; SMF: 1.5T; Sequence: CSE; TR: 600; TE: 9; Matrix: 192×256; FOV: 24cm; Slices: 28; Thickness: 5mm; Inter-slice gap: 0mm.  Confounding factors: none |
| Notes | Funding source: unknown  Conflicts of interest: unknown |

| Bias | Author’s judgment |
| --- | --- |
| Study participation | Low risk |
| Study attrition | Low risk |
| Prognostic factor measurement | Low risk |
| Outcome measurement | Low risk |
| Study confounding | Low risk |
| Statistical analysis and reporting | Low risk |

### Thaler 2015[22]

| Methods | Cross-sectional  Setting: Outpatients  Single-center  Country: Germany |
| --- | --- |
| Participants | 40 participants (37 RRMS, 2 SPMS, and 1 PPMS)  Mean age (SD): 36.9 (10.6)  Sex (M:F): 14/26  Inclusion criteria:   1. diagnosis of MS.   Exclusion criteria: - |
| Prognostic factor | Equipment properties: Scanner: Skyra; SMF: 3T; Sequence: MP-RAGE; TR: 1900; TE: 2.43; Matrix: -; FOV: 25.6cm; Slices: 192; Thickness: 1mm; Inter-slice gap: 0mm.  Confounding factors: unknown |
| Notes | Funding source: not funded  Conflicts of interest: C. Heesen has received lecture honoraria and grants from Biogen, Merck- Serono, Teva, Sanofi Aventis, and Genzyme. |

| Bias | Author’s judgment |
| --- | --- |
| Study participation | High risk |
| Study attrition | Low risk |
| Prognostic factor measurement | Low risk |
| Outcome measurement | Low risk |
| Study confounding | High risk |
| Statistical analysis and reporting | Low risk |

### Truyen 1996[23]

| Methods | Prospective cohort  Setting: Outpatients  Multicenter  Country: Germany  Length of follow-up: 18 months |
| --- | --- |
| Participants | 46 participants (29 RRMS and 17 SPMS)  Median age (range): 35.0 (21 - 53)  Sex (M:F): 18/28  Inclusion criteria:   1. adult age; and 2. established MS diagnosis of either RRMS or SPMS by the Poser criteria.   Exclusion criteria: - |
| Prognostic factor | Equipment properties: Scanner: -; SMF: 0.6T; Sequence: CSE; TR: 450; TE: 28; Matrix: -; FOV: -; Slices: 19; Thickness: 5mm; Inter-slice gap: 1.25mm.  Confounding factors: unknown |
| Notes | Funding source: unknown  Conflicts of interest: unknown |

| Bias | Author’s judgment |
| --- | --- |
| Study participation | Low risk |
| Study attrition | Low risk |
| Prognostic factor measurement | Low risk |
| Outcome measurement | Low risk |
| Study confounding | High risk |
| Statistical analysis and reporting | Low risk |

### Van der Werf 1998[24]

| Methods | Cross-sectional  Setting: Outpatients  Single-center  Country: Netherlands |
| --- | --- |
| Participants | 45 participants (26 RRMS and 19 SPMS)  Mean age (SD): 37.6 (8.4)  Sex (M:F): 17/28  Inclusion criteria:   1. definite MS based on Poser criteria; 2. for RRMS: (a) a history of two or more acute exacerbations in the previous 2 years; (b) have been in a stable neurological state for at least 4 weeks at the time of pre-entry evaluation; (c) have an EDSS of 0–5.0; and 3. for SPMS: (a) have clinically definite secondary progressive multiple sclerosis defined as progressive deterioration of disability for at least 6 months; (b) a deterioration in the EDSS of at least 1 point during the last 2 years, with or without superimposed acute exacerbations following an initial relapsing-remitting course; (c) have an EDSS of 3–6.5.   Exclusion criteria:   1. patients who had an exacerbation in 3 months before assessment; and 2. patients who received any treatment with corticosteroids or ACTH in the 2 months preceding study entry. |
| Prognostic factor | Equipment properties: Scanner: -; SMF: 1.0T; Sequence: CSE; TR: 600; TE: 10; Matrix: -; FOV: -; Slices: -; Thickness: 5mm; Inter-slice gap: -.  Confounding factors: Interferon-beta-1a |
| Notes | Funding source: unknown  Conflicts of interest: unknown |

| Bias | Author’s judgment |
| --- | --- |
| Study participation | Low risk |
| Study attrition | Low risk |
| Prognostic factor measurement | Low risk |
| Outcome measurement | Low risk |
| Study confounding | High risk |
| Statistical analysis and reporting | Low risk |

### van Waesberghe 1998[25]

| Methods | Cross-sectional  Setting: Inpatients and outpatients  Multicenter  Country: Italy and Netherlands |
| --- | --- |
| Participants | 41 participants (8 RRMS and 33 SPMS)  Range of age: 18 - 53  Sex (M:F): 14/27  Inclusion criteria:   1. Diagnosis of MS.   Exclusion criteria: - |
| Prognostic factor | Equipment properties: Scanner: -; SMF: 1.5T; Sequence: CSE; TR: 500; TE: 15; Matrix: -; FOV: -; Slices: -; Thickness: 5mm; Inter-slice gap: -.  Confounding factors: unknown |
| Notes | Funding source: unknown  Conflicts of interest: unknown |

| Bias | Author’s judgment |
| --- | --- |
| Study participation | High risk |
| Study attrition | Low risk |
| Prognostic factor measurement | Low risk |
| Outcome measurement | Unclear risk |
| Study confounding | High risk |
| Statistical analysis and reporting | Low risk |

### Van Walderveen 1999[26]

| Methods | Retrospective cohort  Setting: Outpatients  Single-center  Country: Netherlands  Length of follow-up: 4 years |
| --- | --- |
| Participants | 38 participants (26 RRMS and 12 SPMS)  Median age (range): 33.0 (23 - 52)  Sex (M:F): 18/20  Inclusion criteria:   1. definite MS based on Poser criteria; and 2. at least one month passed after intravenous corticosteroids.   Exclusion criteria:   1. Using immunosuppressives other than infrequent methylprednisolone. |
| Prognostic factor | Equipment properties: Scanner: Technicare; SMF: 0.6T; Sequence: CSE; TR: 2755; TE: 60 and 120; Matrix: -; FOV: -; Slices: 19; Thickness: 5mm; Inter-slice gap: 1.25mm.  Confounding factors: Monoclonal anti-CD4+ cM-T412 antibody |
| Notes | Funding source: Dutch Multiple Sclerosis Society, the Hague, the Netherlands (grant 95-245).  Conflicts of interest: unknown |

| Bias | Author’s judgment |
| --- | --- |
| Study participation | Low risk |
| Study attrition | Low risk |
| Prognostic factor measurement | Low risk |
| Outcome measurement | Low risk |
| Study confounding | High risk |
| Statistical analysis and reporting | Low risk |

### Van Walderveen 2001[27]

| Methods | Cross-sectional  Setting: Outpatients  Single-center  Country: Netherlands |
| --- | --- |
| Participants | 96 participants (52 RRMS, 44 SPMS)  Median age (range): 36 (22 – 58) for RRMS, 43 (30 – 65) for SPMS  Sex (M:F): 34/62  Inclusion criteria:   1. established MS diagnosis of either RRMS, SPMS, or PPMS by the Poser criteria.   Exclusion criteria: - |
| Prognostic factor | Equipment properties: Scanner: Magnetom Impact; SMF: 1.0T; Sequence: CSE; TR: 600; TE: 15; Matrix: -; FOV: -; Slices: 21; Thickness: 5mm; Inter-slice gap: 0.5mm.  Confounding factors: unknown |
| Notes | Funding source: Grant 95-245 from Stichting Vrieden Multiple Sclerosis Research.  Conflicts of interest: unknown |

| Bias | Author’s judgment |
| --- | --- |
| Study participation | Low risk |
| Study attrition | Low risk |
| Prognostic factor measurement | Low risk |
| Outcome measurement | Unclear risk |
| Study confounding | High risk |
| Statistical analysis and reporting | Low risk |

# References

1. Bermel, R.A., et al., *A semiautomated measure of whole-brain atrophy in multiple sclerosis.* Journal of the neurological sciences, 2003. **208**(1-2): p. 57-65.

2. Dastidar, P., et al., *Volumes of brain atrophy and plaques correlated with neurological disability in secondary progressive multiple sclerosis.* Journal of the neurological sciences, 1999. **165**(1): p. 36-42.

3. Dell’Oglio, E., et al., *Quantification of global cerebral atrophy in multiple sclerosis from 3T MRI using SPM: the role of misclassification errors.* Journal of Neuroimaging, 2015. **25**(2): p. 191-199.

4. Dupuy, S.L., et al., *MRI detection of hypointense brain lesions in patients with multiple sclerosis: T1 spin-echo vs. gradient-echo.* European journal of radiology, 2015. **84**(8): p. 1564-1568.

5. Garrido, V., et al. *Whole brain and grey matter atrophy are correlated to long-term disability progression in multiple sclerosis patients*. in *EUROPEAN JOURNAL OF NEUROLOGY*. 2016. WILEY 111 RIVER ST, HOBOKEN 07030-5774, NJ USA.

6. Giorgio, A., et al., *Relevance of hypointense brain MRI lesions for long-term worsening of clinical disability in relapsing multiple sclerosis.* Multiple Sclerosis Journal, 2014. **20**(2): p. 214-219.

7. Giugni, E., et al., *MRI measures and their relations with clinical disability in relapsing-remitting and secondary progressive multiple sclerosis.* Multiple Sclerosis Journal, 1997. **3**(4): p. 221-225.

8. Grimaud, J., et al., *Correlation of magnetic resonance imaging parameters with clinical disability in multiple sclerosis: a preliminary study.* Journal of neurology, 1999. **246**(10): p. 961-967.

9. Khalid, F., et al., *Quantitative MRI analysis of cerebral lesions and atrophy in post-partum patients with multiple sclerosis.* Journal of the neurological sciences, 2018. **392**: p. 94-99.

10. Khan, O., et al., *The relationship between brain MR spectroscopy and disability in multiple sclerosis: 20‐year data from the US glatiramer acetate extension study.* Journal of Neuroimaging, 2017. **27**(1): p. 97-106.

11. Masek, M., et al., *Secondary-progressive form of multiple sclerosis: MRI changes versus clinical status.* Neuro Endocrinol Lett, 2008. **29**(4): p. 461-6.

12. Miki, Y., et al., *Differences between relapsing-remitting and chronic progressive multiple sclerosis as determined with quantitative MR imaging.* Radiology, 1999. **210**(3): p. 769-774.

13. Nijeholt, G., et al., *Brain and spinal cord abnormalities in multiple sclerosis. Correlation between MRI parameters, clinical subtypes and symptoms.* Brain: a journal of neurology, 1998. **121**(4): p. 687-697.

14. O'Riordan, J., et al., *T1 hypointense lesion load in secondary progressive multiple sclerosis: a comparison of pre versus post contrast loads and of manual versus semi automated threshold techniques for lesion segmentation.* Multiple Sclerosis Journal, 1998. **4**(5): p. 408-412.

15. Rovaris, M., et al., *Relevance of hypointense lesions on fast fluid-attenuated inversion recovery MR images as a marker of disease severity in cases of multiple sclerosis.* American journal of neuroradiology, 1999. **20**(5): p. 813-820.

16. Rovaris, M., et al., *Short-term correlations between clinical and MR imaging findings in relapsing-remitting multiple sclerosis.* American Journal of Neuroradiology, 2003. **24**(1): p. 75-81.

17. Sailer, M., et al., *T1 lesion load and cerebral atrophy as a marker for clinical progression in patients with multiple sclerosis. A prospective 18 months follow‐up study.* European journal of neurology, 2001. **8**(1): p. 37-42.

18. Sanfilipo, M.P., et al., *The relationship between whole brain volume and disability in multiple sclerosis: a comparison of normalized gray vs. white matter with misclassification correction.* Neuroimage, 2005. **26**(4): p. 1068-1077.

19. Simon, J.H., et al., *A longitudinal study of T1 hypointense lesions in relapsing MS: MSCRG trial of interferon beta-1a. Multiple Sclerosis Collaborative Research Group.* Neurology, 2000. **55**(2): p. 185-92.

20. Tauhid, S., et al., *Brain MRI lesions and atrophy are associated with employment status in patients with multiple sclerosis.* Journal of neurology, 2015. **262**(11): p. 2425-2432.

21. Tavazzi, E., et al., *Quantitative diffusion weighted imaging measures in patients with multiple sclerosis.* Neuroimage, 2007. **36**(3): p. 746-754.

22. Thaler, C., et al., *T1-thresholds in black holes increase clinical-radiological correlation in multiple sclerosis patients.* PLoS One, 2015. **10**(12): p. e0144693.

23. Truyen, L., et al., *Accumulation of hypointense lesions (" black holes") on T1 spin-echo MRI correlates with disease progression in multiple sclerosis.* Neurology, 1996. **47**(6): p. 1469-1476.

24. Van der Werf, S., et al., *Fatigue in multiple sclerosis: interrelations between fatigue complaints, cerebral MRI abnormalities and neurological disability.* Journal of the neurological sciences, 1998. **160**(2): p. 164-170.

25. van Waesberghe, J., et al., *MR outcome parameters in multiple sclerosis: comparison of surface-based thresholding segmentation and magnetization transfer ratio histographic analysis in relation to disability (a preliminary note).* American journal of neuroradiology, 1998. **19**(10): p. 1857-1862.

26. Van Walderveen, M.A., et al., *Development of hypointense lesions on T1-weighted spin-echo magnetic resonance images in multiple sclerosis: relation to inflammatory activity.* Archives of neurology, 1999. **56**(3): p. 345-351.

27. Van Walderveen, M.A., et al., *Hypointense lesions on T1-weighted spin-echo magnetic resonance imaging: relation to clinical characteristics in subgroups of patients with multiple sclerosis.* Archives of neurology, 2001. **58**(1): p. 76-81.
