# Supplementary material for: Correlation between the clinical disability and T1 hypointense lesions’ volume in cerebral magnetic resonance imaging of multiple sclerosis patients: A systematic review and meta‐analysis
Source: CNS Neurosci Ther. 2021 Oct 3;27(11):1268–80. doi: 10.1111/cns.13734 (PMC8504532; doi:10.1111/cns.13734)
Supplement: Supplementary file 4 — Supplementary Material S4 [file CNS-27-1268-s002.docx]

# Supplementary file D

## Characteristics of excluded studies

[Ordered by study ID]

| Study ID | Reason for exclusion |
| --- | --- |
| Abdelhafeez 2019[1] | Relevant outcome was not assessed. |
| Akaishi 2020[2] | Lesion frequency was reported, not lesion volume. |
| Aymerich 2015[3] | Irrelevant prognostic factor. |
| Bagnato 2011[4] | Lesion frequency was reported, not lesion volume. |
| Barkhof 1999[5] | Relevant outcome was not assessed. |
| Bodini 2009[6] | Relevant outcome was not assessed. |
| Brooks 2012[7] | Relevant outcome was not assessed. |
| Casaccia 2016[8] | Relevant outcome was not assessed. |
| Ciron 2018[9] | Enough data was not provided.  We tried to reach authors for data but were not successful. |
| Collorone 2017[10] | CIS participants consisted more than 15% of the sample. |
| Comini-Frota 2009[11] | Lesion frequency was reported, not lesion volume. |
| Corti 2018[12] | Relevant outcome was not assessed. |
| D'Amico 2016[13] | Lesion frequency was reported not lesion volume. |
| Datta 2015[14] | Enough data was not provided.  We tried to reach authors for data but were not successful. |
| Dekker 2019[15] | All participants were PPMS only. |
| Dell'Oglio 2011[16] | Relevant outcome was not assessed. |
| Essmat 2020[17] | Enough data was not provided.  We tried to reach authors for data but were not successful. |
| Filippi 1998[18] | Enough data was not provided.  We tried to reach authors for data but were not successful. |
| Filippi 2012[19] | Enough data was not provided.  We tried to reach authors for data but were not successful. |
| Filippi 2017[20] | All participants were PPMS only. |
| Fisher 2013[21] | Relevant outcome was not assessed. |
| Freedman 2011[22] | All participants were CIS only. |
| Inglese 2016[23] | All participants were PPMS only. |
| Jeong 2015[24] | Irrelevant participants |
| Kalkers 2001[25] | Enough data was not provided.  We tried to reach authors for data but were not successful. |
| Koch 2015[26] | All participants were PPMS. |
| Lukas 2013[27] | Lesion frequency was reported not lesion volume. |
| Mercado 2020[28] | Relevant outcome was not assessed. |
| Minneboo 2008[29] | Enough data was not provided.  We tried to reach authors for data but were not successful. |
| Mitrovic 2018[30] | Irrelevant participants |
| Molyneux 1998[31] | Relevant outcome was not assessed. |
| Moreira 2010[32] | Enough data was not provided.  We tried to reach authors for data but were not successful. |
| Nakamura 2016[33] | Relevant outcome was not assessed. |
| Nash 2016[34] | Relevant outcome was not assessed. |
| Nittner-Marszalska 2011[35] | Lesion frequency was reported, not lesion volume. |
| Ontaneda 2017[36] | PPMS participants consisted more than 15% of the sample. |
| Oreja-Guevara 2013[37] | Relevant outcome was not assessed. |
| Paolillo 1999[38] | Enough data was not provided.  We tried to reach authors for data but were not successful. |
| Portaccio 2009[39] | Irrelevant prognostic factor. |
| Prosperini 2011[40] | Specific brain region studied |
| Razzolini 2016[41] | Enough data was not provided.  We tried to reach authors for data but were not successful. |
| Razzolini 2017[42] | Enough data was not provided.  We tried to reach authors for data but were not successful. |
| Roosendaal 2011[43] | Enough data was not provided.  We reached authors for data but didn’t receive any. |
| Ruberte 2016[44] | Relevant outcome was not assessed. |
| Rudick 2013[45] | Enough data is not provided.  We tried to reach authors for data but were not successful. |
| Schlaeger 2015[46] | Irrelevant prognostic factor. |
| Sempere 2011[47] | Enough data was not provided.  We tried to reach authors for data but were not successful. |
| Stevenson 1999[48] | PPMS participants consisted more than 15% of the sample. |
| Stevenson 2000[49] | PPMS participants consisted more than 15% of the sample. |
| Stromillo 2015[50] | Irrelevant prognostic factor and irrelevant participants. |
| Teodorescu 2014[51] | All participants were PPMS. |
| Ukkonen 2003[52] | All participants were PPMS. |
| Vaneckova 2002[54] | Relevant outcome was not assessed. |
| van Walderveen 1995[53] | Relevant outcome was not assessed. |
| Varhaug 2017[55] | Relevant outcome was not assessed. |
| Vermersch 2017[56] | Lesion frequency was reported, not lesion volume. |
| Weier 2013[57] | Relevant outcome was not assessed. |
| Zakaria 2016[58] | Enough data was not provided.  We reached authors for data but didn’t receive any. |
| Zivadinov 2003[59] | Enough data was not provided.  We tried to reach authors for data but were not successful. |

# References

References to studies excluded from this review

1. Abdelhafeez, M.A., et al., *Magnetic resonance imaging markers of disability in Egyptian multiple sclerosis patients.* Mult Scler Relat Disord, 2019. **36**: p. 101417.

2. Akaishi, T., et al., *Number of MRI T1-hypointensity corrected by T2/FLAIR lesion volume indicates clinical severity in patients with multiple sclerosis.* PLoS One, 2020. **15**(4): p. e0231225.

3. Aymerich, F.X., et al., *Longitudinal MRI study to measure cervical cord atrophy in multiple sclerosis patients.* Multiple Sclerosis, 2015. **23**(11): p. 472-473.

4. Bagnato, F., et al., *Lesions by tissue specific imaging characterize multiple sclerosis patients with more advanced disease.* Multiple Sclerosis Journal, 2011. **17**(12): p. 1424-1431.

5. Barkhof, F., *MRI in multiple sclerosis: correlation with expanded disability status scale (EDSS).* Mult Scler, 1999. **5**(4): p. 283-6.

6. Bodini, B., et al., *Spatial localisation of lesions predicts clinical deterioration in primary progressive multiple sclerosis: A 10-year multi-centre study.* Multiple Sclerosis, 2009. **15**(9): p. S11.

7. Brooks, J.B.B., et al., *A role for the Blink Reflex test in the diagnosis and progression of disease assessment in multiple sclerosis.* Multiple Sclerosis, 2012. **18**(12): p. 1821.

8. Casaccia, P., et al., *Epigenomic changes in monocytes from RRMS patients with high body mass index and related preclinical animal models.* Multiple Sclerosis, 2016. **22**: p. 502-503.

9. Ciron, J., et al., *Treatment of progressive MS with MD1003 (high dose pharmaceutical grade biotin): Real-world evidence.* Multiple Sclerosis Journal, 2018. **24**(2): p. 693.

10. Collorone, S., et al., *Alterations in individual cortical networks of CIS patients: A longitudinal multi-centre MAGNIMS study.* Multiple Sclerosis Journal, 2017. **23**(3): p. 550-551.

11. Comini-Frota, E.R., et al., *Association of CXCL10 serum levels and other chemokines with gender, inflammatory activity and immunomodula-tory treatment regimen in multiple sclerosis patients.* Multiple Sclerosis, 2009. **15**(9): p. S198.

12. Corti, L., et al., *Late clinical activity in long-standing non-active multiple sclerosis patients.* Multiple Sclerosis Journal, 2018. **24**(2): p. 341-342.

13. D'Amico, E., et al., *Lateral and escalation therapy in relapsing-remitting multiple sclerosis: a comparative study.* J Neurol, 2016. **263**(9): p. 1802-9.

14. Datta, S., et al., *Regional gray matter atrophy in relapsing remitting multiple sclerosis: baseline analysis of multi-center data.* Mult Scler Relat Disord, 2015. **4**(2): p. 124-36.

15. Dekker, I., et al., *Predicting clinical progression in multiple sclerosis after 6 and 12 years.* Eur J Neurol, 2019. **26**(6): p. 893-902.

16. Dell'Oglio, E., et al., *A segmentation pipeline to assess global cerebral grey-matter atrophy in multiple sclerosis from 3T MRI.* Multiple Sclerosis, 2011. **17**(10): p. S151-S152.

17. Essmat, A., et al., *Novel Imaging Techniques in Detection of Progressive Multiple Sclerosis.* Multiple Sclerosis and Related Disorders, 2020. **37**.

18. Filippi, M., et al., *Quantitative volumetric analysis of brain magnetic resonance imaging from patients with multiple sclerosis.* J Neurol Sci, 1998. **158**(2): p. 148-53.

19. Filippi, M., et al., *Grey-matter damage predicts the accumulation of disability and cognitive impairment 13 years later in patients with multiple sclerosis.* Journal of Neurology, 2012. **259**(1): p. S149-S150.

20. Filippi, M., et al., *Earlier prognostication in primary progressive multiple sclerosis using MRI: A 15-year longitudinal study.* European Journal of Neurology, 2017. **24**: p. 43.

21. Fisher, E., J.C. Lee, and R. Rudick, *Temporal patterns of brain atrophy in individual multiple sclerosis patients.* Neurology, 2013. **80**(1).

22. Freedman, M., et al., *Predictors of disease activity in CIS patients treated with IFNB-1b in the BENEFIT study.* Multiple Sclerosis, 2011. **17**(10): p. S16-S17.

23. Inglese, M., et al., *Cerebellar volume as an outcome measure in therapeutic trials of primary progressive multiple sclerosis.* Multiple Sclerosis, 2016. **22**: p. 198-199.

24. Jeong, I.H., et al., *Comparison of myelin water fraction values in periventricular white matter lesions between multiple sclerosis and neuromyelitis optica spectrum disorder: A preliminary report.* Multiple Sclerosis, 2015. **23**(11): p. 478.

25. Kalkers, N.F., et al., *Optimizing the association between disability and biological markers in MS.* Neurology, 2001. **57**(7): p. 1253-8.

26. Koch, M.W., et al., *MRI measures and disability progression in PPMS: Analysis of the PROMiSe clinical trial dataset.* Multiple Sclerosis, 2015. **23**(11): p. 46.

27. Lukas, C., et al., *Relevance of spinal cord abnormalities to clinical disability in multiple sclerosis: MR imaging findings in a large cohort of patients.* Radiology, 2013. **269**(2): p. 542-52.

28. Mercado, V., et al., *Multiple Sclerosis in a Multi-Ethnic Population in Houston, Texas: A Retrospective Analysis.* Biomedicines, 2020. **8**(12).

29. Minneboo, A., et al., *Predicting short-term disability progression in early multiple sclerosis: added value of MRI parameters.* J Neurol Neurosurg Psychiatry, 2008. **79**(8): p. 917-23.

30. Mitrovic, T., et al., *MRI brain T1 gray matter/white matter contrast in multiple sclerosis versus migraine using magnetization prepared rapid gradient echo (MPRAGE) sequences.* Multiple Sclerosis Journal, 2018. **24**(2): p. 16-17.

31. Molyneux, P., et al., *Correlations between monthly enhanced MRI lesion rate and changes in T2 lesion volume in multiple sclerosis.* Annals of neurology, 1998. **43**(3): p. 332-339.

32. Moreira, F., et al., *Pattern of brain activation in multiple sclerosis patients with fatigue: A functional magnetic resonance study.* Multiple Sclerosis, 2010. **16**(10): p. S105-S106.

33. Nakamura, K., et al., *Quantitative analysis of normal-appearing brain tissue in MS using magnetic resonance fingerprinting.* Multiple Sclerosis, 2016. **22**: p. 526-527.

34. Nash, R., et al., *Five-year outcomes of halt-MS: High-dose immunosuppressive therapy and autologous hematopoietic cell transplantation for severe relapsing-remitting multiple sclerosis.* Bone Marrow Transplantation, 2016. **51**: p. S61-S62.

35. Nittner-Marszalska, M., et al., *Wasp venom immunotherapy in a patient with multiple sclerosis.* Allergy: European Journal of Allergy and Clinical Immunology, 2011. **66**: p. 439.

36. Ontaneda, D., et al., *Vitamin D levels and MS features in progressive multiple sclerosis.* Multiple Sclerosis Journal, 2017. **23**(3): p. 207.

37. Oreja-Guevara, C., et al., *Study of magnetisztion transfer MRI in clinically isolated syndromes (CIS).* Journal of Neurology, 2013. **260**: p. S178.

38. Paolillo, A., et al., *Quantitative MRI in patients with secondary progressive MS treated with monoclonal antibody Campath 1H.* Neurology, 1999. **53**(4): p. 751-7.

39. Portaccio, E., et al., *Neuropsychological and MRI measures predict short-term evolution in benign multiple sclerosis.* Neurology, 2009. **73**(7): p. 498-503.

40. Prosperini, L., et al., *The relationship between infratentorial lesions, balance deficit and accidental falls in multiple sclerosis.* J Neurol Sci, 2011. **304**(1-2): p. 55-60.

41. Razzolini, L., et al., *Neuropsychological features can help in predicting disease evolution in benign multiple sclerosis patients: A 12 year study.* Multiple Sclerosis, 2016. **22**: p. 110.

42. Razzolini, L., et al., *Cognitive impairment can help to predict long-term disease course in benign multiple sclerosis patients: A 12 year follow-up study.* Multiple Sclerosis Journal, 2017. **23**(3): p. 117.

43. Roosendaal, S.D., et al., *Grey matter volume in a large cohort of MS patients: relation to MRI parameters and disability.* Mult Scler, 2011. **17**(9): p. 1098-106.

44. Ruberte, E., et al., *Lateral Ventricle Volume change is associated with neurological and cognitive disability in multiple sclerosis: A 5-years follow-up study using an automated lateral ventricle segmentation algorithm.* Swiss Medical Weekly, 2016. **146**: p. 88S.

45. Rudick, R., J.C. Lee, and E. Fisher, *“Mild multiple sclerosis”: Results from an 11 year observational study of brain atrophy.* Neurology, 2013. **80**(1).

46. Schlaeger, R., et al., *Spinal cord gray matter atrophy - A biomarker for MS progression.* Schweizer Archiv fur Neurologie und Psychiatrie, 2015. **166**: p. 33.

47. Sempere, A.P., et al., *Longitudinal study of retinal nerve fibre layer thickness in multiple sclerosis.* Multiple Sclerosis, 2011. **17**(10): p. S492.

48. Stevenson, V.L., et al., *Primary and transitional progressive MS: a clinical and MRI cross-sectional study.* Neurology, 1999. **52**(4): p. 839-45.

49. Stevenson, V.L., et al., *One year follow up study of primary and transitional progressive multiple sclerosis.* J Neurol Neurosurg Psychiatry, 2000. **68**(6): p. 713-8.

50. Stromillo, M.L., et al., *Brain atrophy rates and sustained disability over 10 years in multiple sclerosis.* European Journal of Neurology, 2015. **22**: p. 214.

51. Teodorescu, R., et al., *Relationship between spinal cord atrophy and brain cortical lesions in primary-progressive ms.* Neurology, 2014. **82**(10).

52. Ukkonen, M., et al., *Volumetric quantitation by MRI in primary progressive multiple sclerosis: volumes of plaques and atrophy correlated with neurological disability.* Eur J Neurol, 2003. **10**(6): p. 663-9.

53. van Walderveen, M.A., et al., *Correlating MRI and clinical disease activity in multiple sclerosis: relevance of hypointense lesions on short-TR/short-TE (T1-weighted) spin-echo images.* Neurology, 1995. **45**(9): p. 1684-90.

54. Vaneckova, M., et al., *New trends of MR imaging in multiple sclerosis. Our experience with MR volumometry using local software.* Ceska Radiologie, 2002. **56**(6): p. 327-330.

55. Varhaug, K., et al., *Serum neurofilament light chain predicts disease activity in relapsing remitting multiple sclerosis.* Multiple Sclerosis Journal, 2017. **23**(3): p. 589-590.

56. Vermersch, P., et al., *A comparison of multiple sclerosis disease activity after discontinuation of fingolimod and placebo.* Mult Scler J Exp Transl Clin, 2017. **3**(3): p. 2055217317730096.

57. Weier, K., et al., *Six-year follow-up of a case series with non-communicating syringomyelia in multiple sclerosis.* Eur J Neurol, 2013. **20**(3): p. 578-83.

58. Zakaria, M.F., et al., *Clinical and radiological patterns of multiple sclerosis among a sample of Egyptian patients.* Multiple Sclerosis, 2016. **22**(6): p. NP17.

59. Zivadinov, R., et al., *Normalized regional brain atrophy measurements in multiple sclerosis.* Neuroradiology, 2003. **45**(11): p. 793-8.
