# Supplementary material for: Correlation between the clinical disability and T1 hypointense lesions’ volume in cerebral magnetic resonance imaging of multiple sclerosis patients: A systematic review and meta‐analysis
Source: CNS Neurosci Ther. 2021 Oct 3;27(11):1268–80. doi: 10.1111/cns.13734 (PMC8504532; doi:10.1111/cns.13734)

**Supplementary file E**

**Subgroups and sensitivity analyses**

**Analysis 1. Subgroup analyses based on MS category (RRMS or SPMS)**


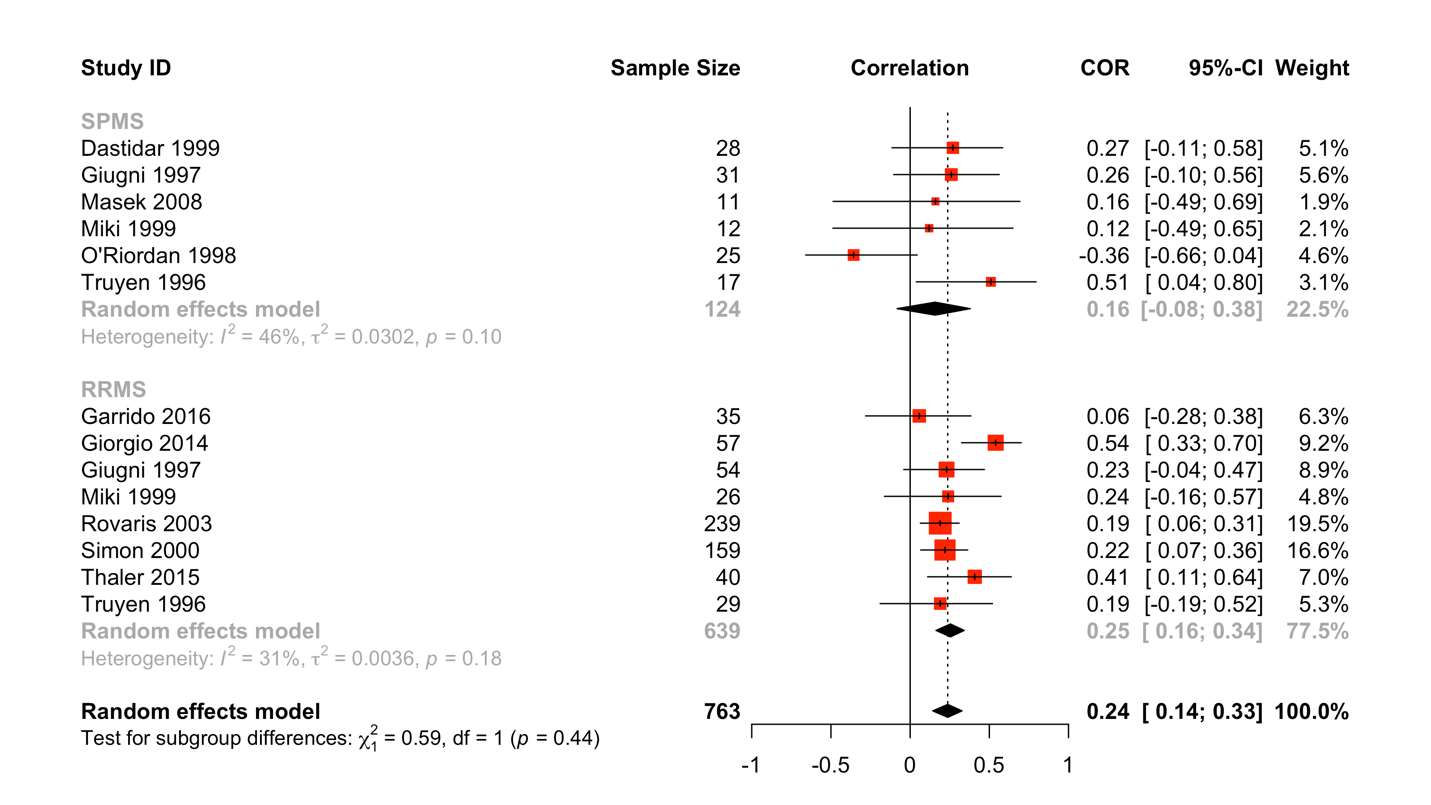


**Analysis 2. Subgroup analyses based on diagnostic criteria (Poser or McDonald)**

**
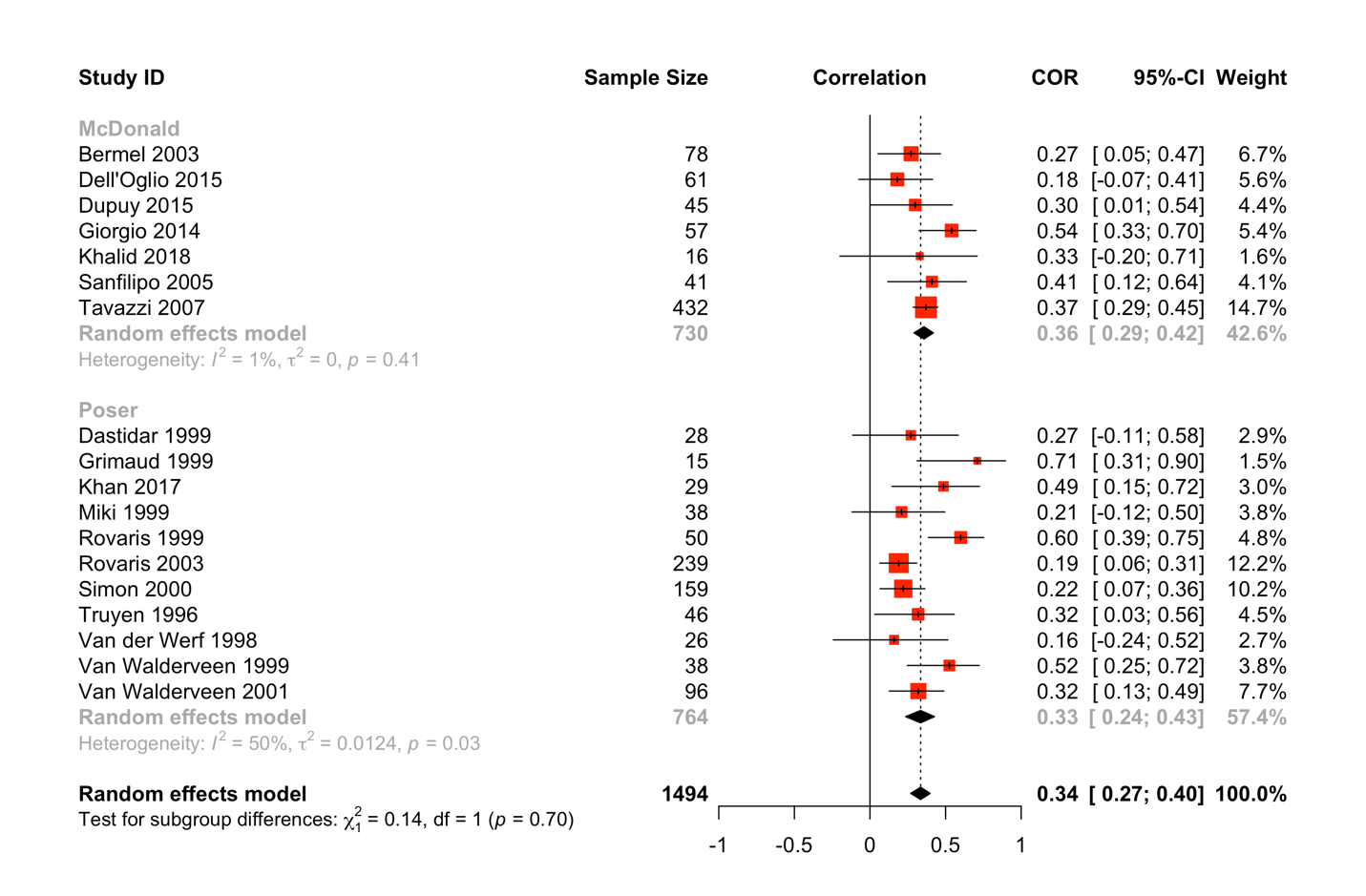
**

**Analysis 3. Subgroup analyses based on SMF (≥1.5T or <1.5T)**

**
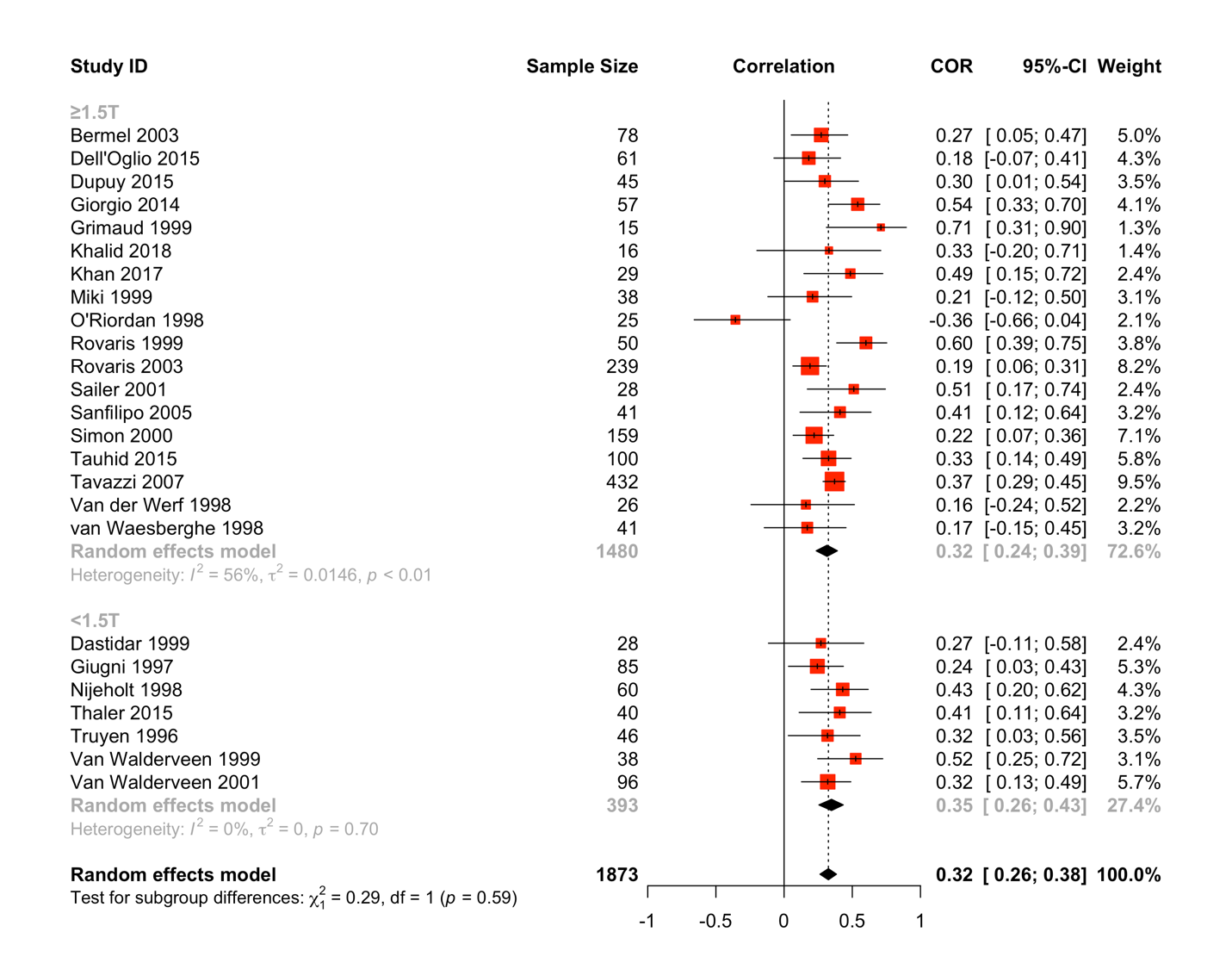
**

**Analysis 4. Sensitivity analysis on studies with a large sample size (>90)**


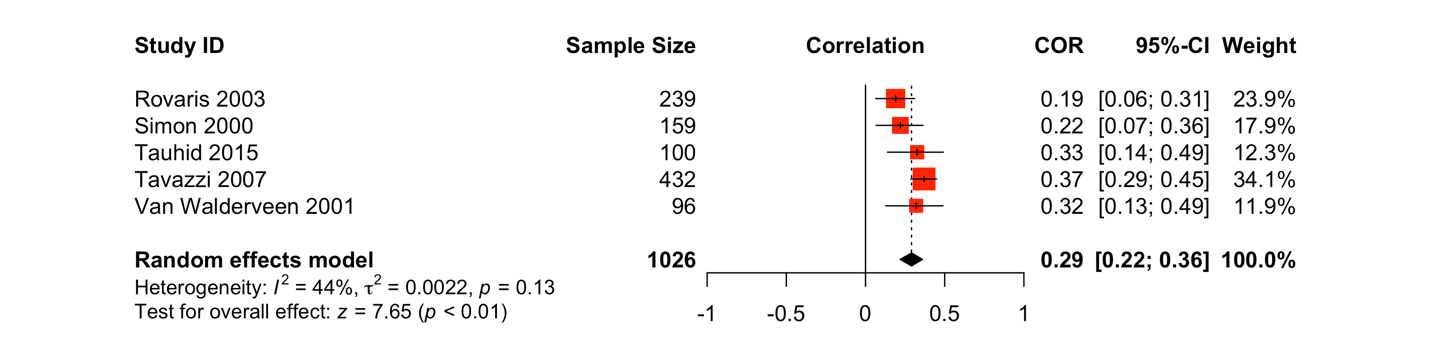


**Analysis 5. Sensitivity analysis on studies with the high/unclear risk of bias in the Participation domain**


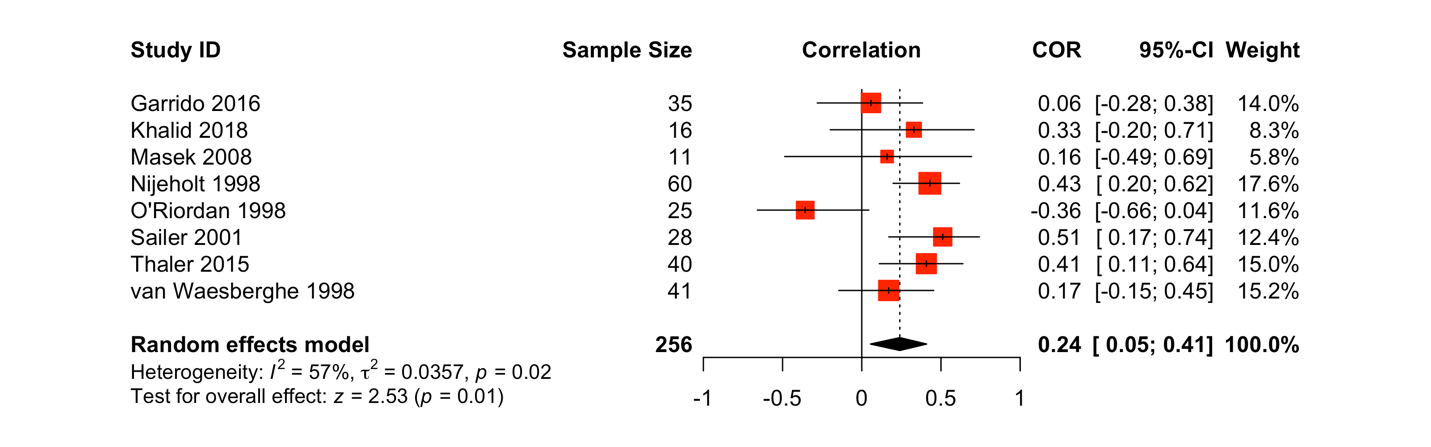


**Analysis 6. Sensitivity analysis on studies with the high/unclear risk of bias in the Outcome measurement domain**


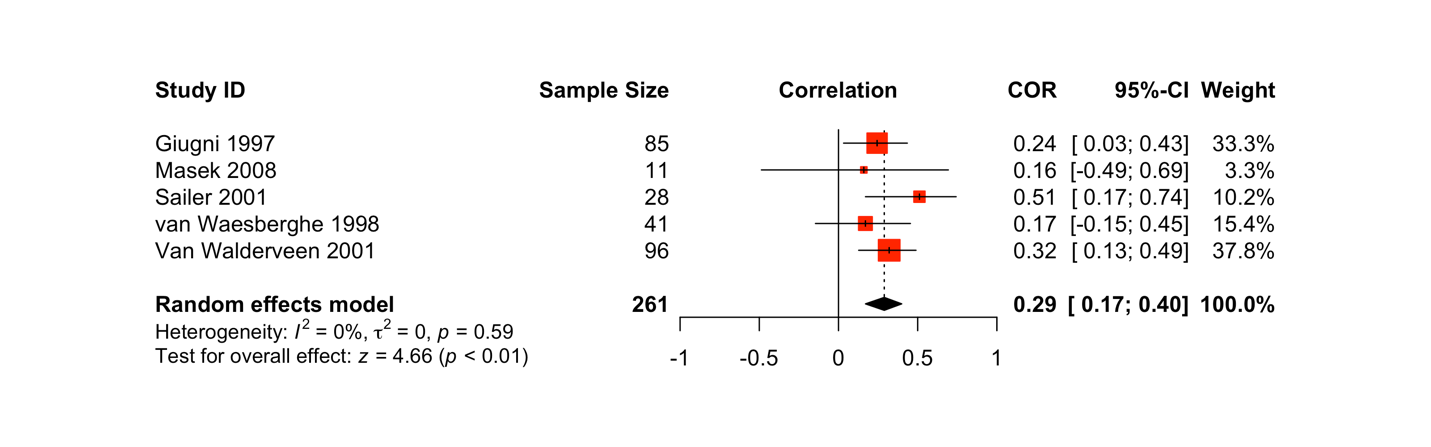


**Analysis 7. Sensitivity analysis on studies with the high/unclear risk of bias in the Study confounding domain**


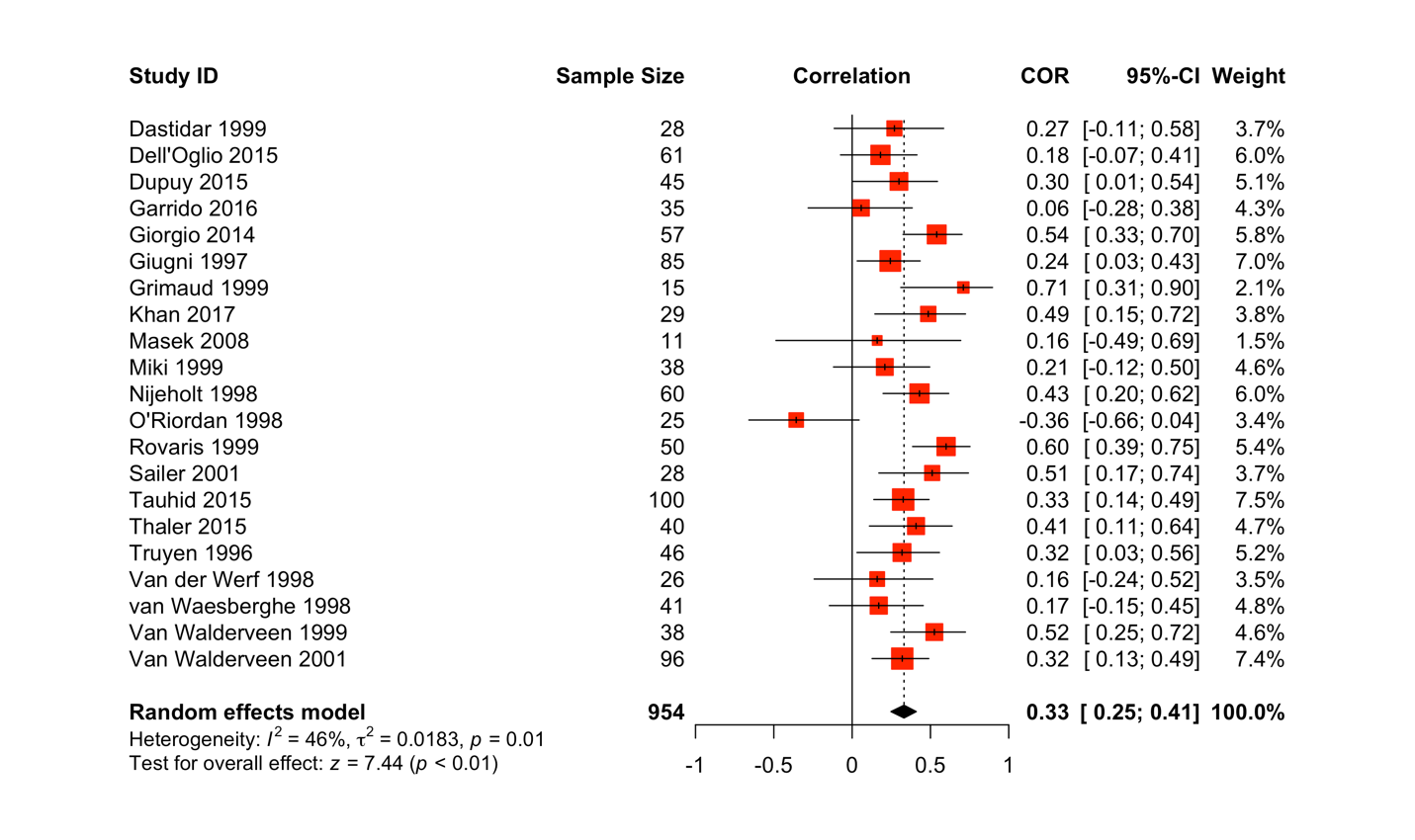

Supplement: Supplementary file 5 — Supplementary Material S5 [file CNS-27-1268-s006.docx]
